# Engineering a two-gene system to operate as a highly sensitive biosensor or a sharp switch upon induction with β-estradiol

**DOI:** 10.1038/s41598-022-26195-x

**Published:** 2022-12-16

**Authors:** Tian Zhou, Zhiying Liang, Mario Andrea Marchisio

**Affiliations:** 1 School of Pharmaceutical Science and Technology, Tianjin University, 92 Weijin Road, Tianjin, 300072 China; 2 School of Life Science and Technology, Harbin Institute of Technology, 2 Yikuang Street, Harbin, 150080 China

**Keywords:** Biotechnology, Molecular biology

## Abstract

The human estrogen receptor has been used for about thirty years, in the yeast *S. cerevisiae*, as a component of chimeric transcription factors. Its ligand, β-estradiol, permits to control the protein translocation into the nucleus and, as a consequence, the expression of the gene(s) targeted by the synthetic transcription factor. Activators that are orthogonal to the yeast genome have been realized by fusing the human estrogen receptor to an activation and a DNA-binding domain from bacteria, viruses, or higher eukaryotes. In this work, we optimized the working of a β-estradiol-sensing device—in terms of detection range and maximal output signal—where the human estrogen receptor is flanked by the bacterial protein LexA and either the strong VP64 (from herpes simplex virus) or the weaker B42 (from *E. coli*) activation domain. We enhanced the biosensor performance by thoroughly engineering both the chimeric activator and the reporter protein expression cassette. In particular, we constructed a synthetic promoter—where transcription is induced by the chimeric activators—based on the core sequence of the yeast *CYC1* promoter, by tuning parameters such as the length of the 5′ UTR, the distance between adjacent LexA binding sites (operators), and the spacing between the whole operator region and the main promoter TATA box. We found a configuration that works both as a highly sensitive biosensor and a sharp switch depending on the concentration of the chimeric activator and the strength of its activation domain.

## Introduction

Synthetic Biology is a new branch of life science that aims at reengineering living cells such that they carry out new, precise functions^1^. Its origin can be traced back to the first two synthetic gene circuits published in January 2000^2,3^. Among the circuits that followed, biosensors became the object of many research works due to their numerous possible applications^4^. Biosensors have been engineered to respond to several different inputs, e.g., heavy metals^5,6^, metabolites^7^, organophosphates^8^ 2,4-dinitrotoluene^9^, intracellular xylose^10^, and arsenic^11^.

In yeast Synthetic Biology, the β-estradiol biosensor has been engineered in various configurations and used, mainly, as a tool to control the synthesis of endogenous and synthetic genes. β-estradiol diffuses through the yeast membrane and does not provoke, per se, toxic effects unless its concentration becomes much higher than 1000 nM^12^. In the Eighties of the last century, Ma and Ptashne^13^ realized a collection of yeast chimeric activators that combined either GAL4 or LexA DNA-binding domain (DBD) with one of 15 bacterial activation domains (ADs)—among which there was also B42 that we used in this work. The chimeric activator library was later extended with the inclusion of more bacterial ADs (e.g., B112)^14^. In 1993, Louvion et al*.*^12^ engineered the first β-estradiol-inducible chimeric activator (later referred to as GEV) by merging GAL4 DBD with the hormone binding domain of the human estrogen receptor—HBD(hER), where β-estradiol binds—and the strong VP16 AD from the herpes simplex virus type 1^15^. GEV permitted to activate any galactose-inducible promoter in glucose-containing media, where the growth of *S. cerevisiae* cells is faster. More recently, Mclsaac et al*.*^16^ turned GEV into Z_3_EV by replacing GAL4 DBD with the zinc finger protein Zif268 DBD. Ottoz et al*.*^17^, in contrast, modified GEV by using LexA as a DBD and tested four ADs, among which B112 was the most performant. Both works aimed at constructing a system for the control of gene expression in *S. cerevisiae* without interfering with the original molecular processes that take place in the cells. In Synthetic Biology, such a system is termed *orthogonal* to the host cell. Finally, Dossani et al*.*^18^ built a large library of hybrid promoters consisting of core yeast promoters preceded by a variable number of LexA binding site (operators). Each promoter was activated by LexA-HBD(hER)-VP16 at low (up to 100 nM) concentrations of β-estradiol.

**Figure 1:**
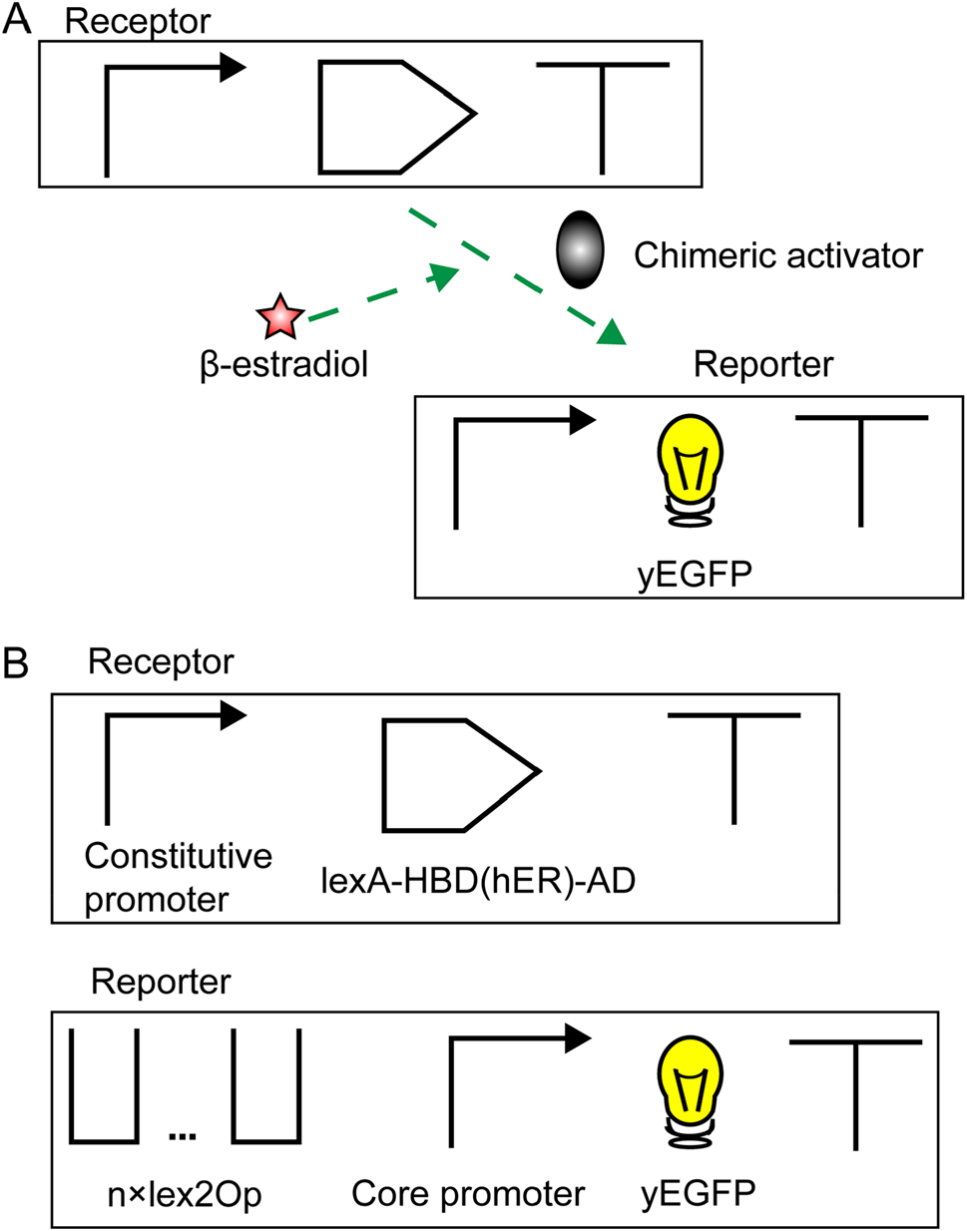
The β-estradiol biosensor. (**A**) Biosensor scheme. The dashed arrow means that the activator protein cannot bind and activate the target promoter in the absence of its inducer. (**B**) Structure of the two TUs in our β-estradiol biosensors. The number (n) of lex2Op used in this work goes from 1 to 8.

A complete β-estradiol biosensor is divided into two components: the receptor and the reporter^19^. They are transcription units (TUs), i.e., DNA sequences made of a promoter, a coding region, and a terminator. The receptor expresses constitutively a chimeric activator hosting HBD(hER). The reporter produces a fluorescent protein only in the presence of β-estradiol (see Fig. 1A).

Despite all the implementations mentioned above, a common way to characterize a β-estradiol biosensor has not been established yet. In^16,17^, important parameters are the basal fluorescence (i.e., the fluorescence expressed by the biosensor in the absence of β-estradiol) and the maximal fluorescence reached by the circuit—which quantifies how strongly a gene can be expressed under β-estradiol induction. Basal fluorescence shall be as close as possible to zero, whereas the maximal fluorescence should equal or even overcome that of the strongest yeast constitutive promoter (i.e., the *GPD* promoter). In^18^, these two quantities are merged into a single one, the inducibility, that roughly corresponds to the ON/OFF ratio.

In order to obtain both low basal fluorescence and high response from the same β-estradiol biosensor, its components shall be balanced carefully. In the receptor part, the strength of promoter and AD should be chosen to avoid the overexpression of the chimeric activator, which would likely cause toxicity effects^20^. The reporter shall host a reasonably weak core promoter preceded by a thoroughly engineered operator cassette. Here, several parameters shall be adjusted (e.g., operator number, distance between two adjacent operators, separation from the TATA box) to achieve a good trade-off between basal and maximal fluorescence level.

As a sensing device, however, a β-estradiol biosensor should be also characterized by its sensitivity and tolerance to the input, which permit to define the detection range over which the biosensor works reliably. None of the β-estradiol biosensors in the literature was evaluated according to these parameters, as if its original function (detecting β-estradiol) had been forgotten. In this work, we propose a general way to assess the performance of a β-estradiol biosensor. This requires estimating: (1) the detectivity (D), i.e., the minimal β-estradiol concentration that is unequivocally detected by the biosensor; (2) the tolerance, i.e., the maximal concentration of β-estradiol at which the biosensor activity is not damaged by toxicity effects; (3) the detection range, which goes from D to T; and (4) the maximal output (usually fluorescence) level, which tells us if the biosensor can be used to enhance the expression of a target gene up to a desired level.

Taking inspiration from^17^, we engineered deeply both receptor and reporter in order to find out criteria to optimize the working of the β-estradiol (and potentially other) biosensor(s). Like in^17^, also in our work the receptor includes the full LexA bacterial protein as a DBD. LexA binds, as a dimer, a 41-nt-long operator that is organized in two halves of 20 nt separated by a single base pair. Thus, the lex operator is usually referred to as lex2Op. We fused LexA to HBD(hER) and, separately, four different ADs. In the absence of β-estradiol, the heat shock protein 90 (Hsp90) sequesters the activator in the cytoplasm upon binding HBD(hER). In contrast, β-estradiol—when present in the cell culture—docks to HBD(hER) and prevents any further interactions with Hsp90, such that the activator translocates into the nucleus^19^ (see Fig. S1).

The performance of the whole biosensor depends on three main components: (1) the constitutive promoter upstream of the activator; (2) the synthetic promoter upstream of the fluorescent protein; and (3) the AD at the C-terminus of the chimeric activator, which recruits RNA polymerase II to the activated promoter.

We engineered, first, yeast strains for the expression of yEGFP (yeast enhanced green fluorescent protein). They contained different weak promoters, based on variants of the *CYC1* core promoter^21^, preceded by lex2Op sites in a variable number. On these strains, we constructed overall ten types of β-estradiol biosensors.

The initial configuration performed poorly by returning a low output signal in the presence of β-estradiol. We improved and, finally, optimized the biosensor design by making several changes on the promoter of the reporter part. We modified the number of lex2Op, their reciprocal distance, the distance between the complete operator cassette and the main TATA box, and the length of the 5′UTR. Moreover, we tested three constitutive promoters and four ADs of different strength on the receptor side, in order to tune the expression both of the chimeric activator and yEGFP.

This work of deep engineering led us to formulate criteria to optimize the β-estradiol biosensor and convert it into a switch responding to the same hormone.

## Results and discussion

We engineered, overall, ten kinds of β-estradiol biosensors organized in a receptor and a reporter TU. The receptor encoded for the chimeric activator LexA-HBD(hER)-AD, where AD was one among: VP16, VP64 (i.e., the fusion of four VP16 units), mDR521-805—from the mouse dioxin receptor protein^22^—and B42. The reporter expressed yEGFP upon activation of the synthetic promoter that combined a fragment of the core yeast constitutive *CYC1* promoter and a variable quantity of lex2Op sites (see Fig. 1B). We carried out a deep engineering work to understand which features were essential either to enhance the output signal or to detect very low concentrations of the input.

There are various ways to evaluate how a biosensor works. Since it represents a YES (or buffer) Boolean gate, the most straightforward parameter would be the gain (i.e., the ON/OFF ratio) or the signal separation (i.e., the difference between the one and the zero output). However, each biosensor configuration was characterized via a titration curve, i.e., the fluorescence output was measured for twelve concentrations of β-estradiol (from 0.98 to 2000 nM—a range over which β-estradiol is not toxic, per se, to the yeast cells) and in the absence of the hormone. Therefore, other quantities appeared more significative: (1) the basal fluorescence, i.e., the fluorescence expressed in the absence of β-estradiol, which quantifies the biosensor leakage; (2) the detectivity (D), that corresponds to the minimal concentration of β-estradiol that is sensed by a biosensor, i.e., the smallest concentration of the input that induces an output signal at least twofold higher than the noise—where the noise is the highest between the background fluorescence (i.e., the mean fluorescence of the empty chassis—byMM2: 52.45 ± 1.86 A.U.) and the basal fluorescence, as defined previously. It should be noted, though, that the background fluorescence has been subtracted from all fluorescence values reported in this work such that, to determine D, we should look for the concentration of β-estradiol that induced an output signal either bigger than (or equal to) 52.45 A.U. or twice as high as the basal fluorescence; (3) the tolerance, which represents the maximal concentration of β-estradiol that is detected unambiguously before the occurrence of toxicity effects; (4) the detection range, which is the interval in the concentration of β-estradiol delimited by the detectivity and the tolerance; (5) the maximal fluorescence signal—which we used as a reference to quantify the effects on the biosensor due to changes in one (or more) of its elements.

### Minimizing the basal fluorescence by employing the weak promoter *truncated_pCYC1min* in the reporter. Maximizing the expression of the chimeric activator from the receptor to enhance fluorescence in the presence of β-estradiol

Biosensor 1 design: pGPD-LexA-HBD(hER)-AD and 7×lex2Op(2, 37-TATA_-52_)-truncated_pCYC1min (see Fig. S6).

**Figure 2:**
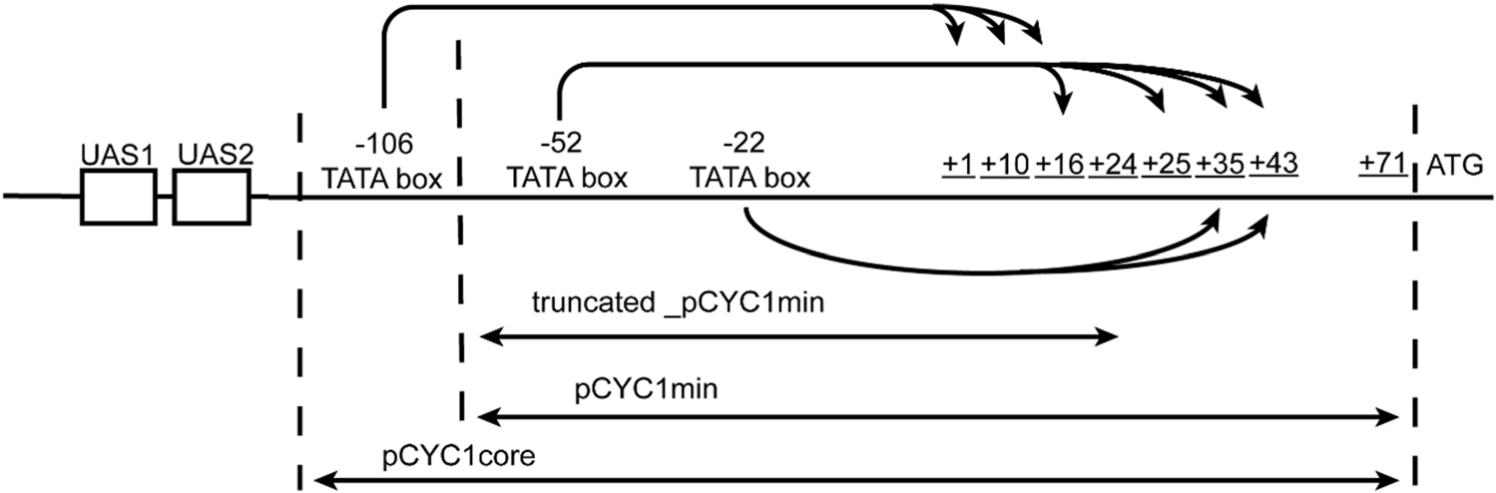
Scheme of *CYC1* promoter and its shorter derivatives used in this work. The number from + 1 to + 43 represent different TSSs (transcription start sites). Arrows with multiple tips on the right indicate all TSSs that are activated by the same TATA box (usually, they lie from 40 up to 120 nt downstream of the TATA box).

Our initial target was a weak variant of the *CYC1* core promoter, where the TATA box starting at -106 (TATA_-106_) was removed and the 5′UTR shortened from 71 to 24 nt (see Fig. 2). This promoter, termed *truncated_pCYC1min*, was characterized by a very low fluorescence expression (79.34 A.U.)^23,24^ corresponding to 2.44% of that of pCYC1min (and 0.43% of that of pGPD—see Table S1). To turn it into an activated promoter, we placed seven copies of lex2Op upstream of its sequence, realizing the topology: 7×lex2Op(2, 37-TATA_-52_)-truncated_pCYC1min. The first number within the brackets indicates that adjacent lex2Op were separated by two nucleotides (always GT in this case), whereas the second notation means that the whole operator cassette was placed 37 nt upstream of the TATA box starting at position -52 (TATA_-52_).

We expressed the chimeric activator in high amount via the strong *GPD* promoter (pGPD) and tested four ADs: the strong V16, VP64, and mDR521-805 together with the weak B42 (in the rest of this section, we will use the AD name to refer to the whole biosensor).

**Figure 3:**
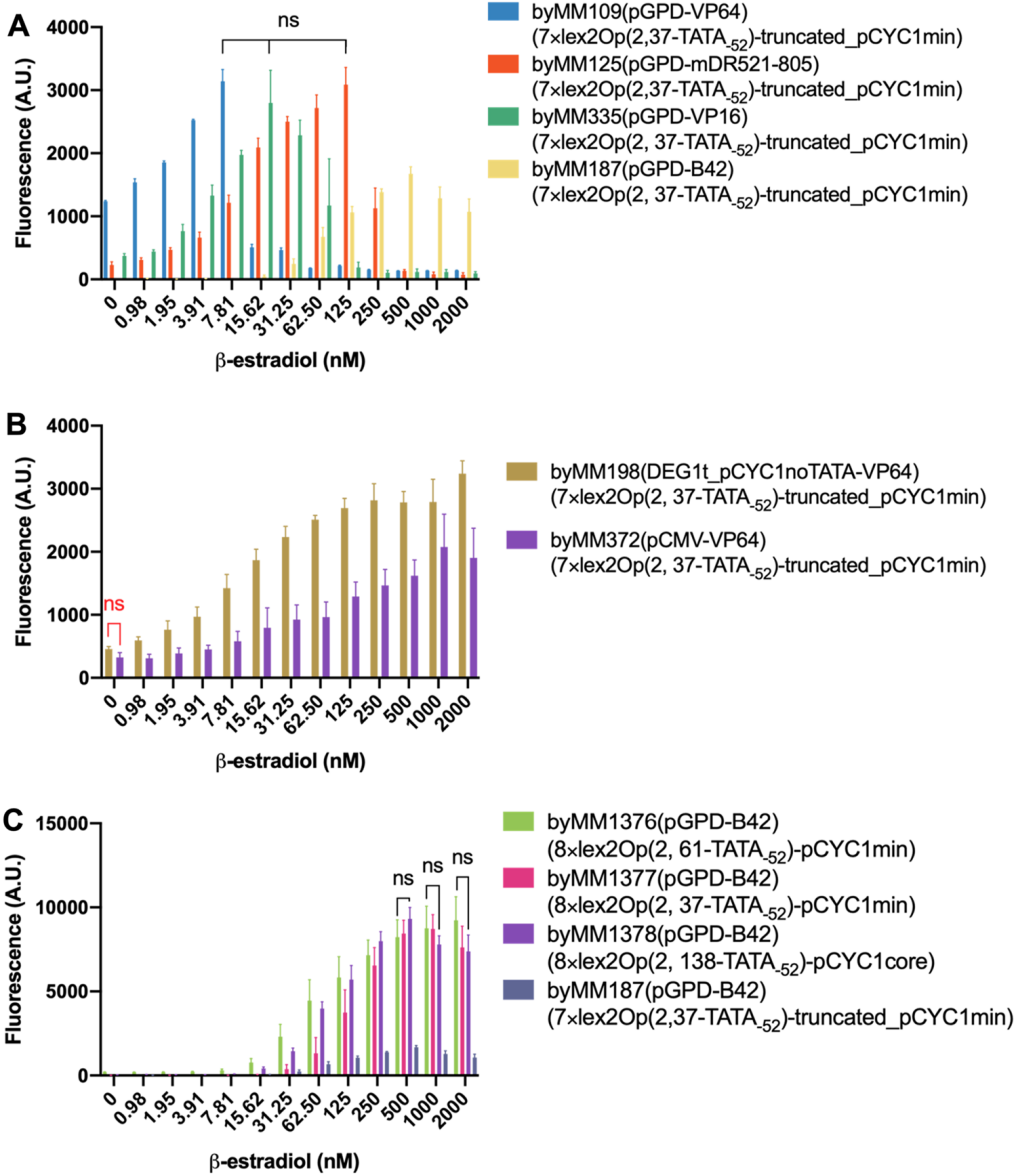
Response of biosensors 1–3 to increasing concentrations of β-estradiol. (**A**) Biosensors 1. The receptor TU employs the strong pGPD, whereas the AD is variable. The promoter in the reporter TU is characterized by seven copies of lex2Op—spaced by only 2 nt—that lie 37 nt upstream of the very weak truncated_pCYC1min. Despite the high number of lex2Op and the presence of strong ADs in large amount, our synthetic promoter was poorly activated. The maximal fluorescence level of byMM1376-byMM1378 are statistically equivalent (ns: one-way ANOVA; p-value = 0.4843). (**B**) Biosensors 2. DEG1t_pCYC1noTATA and pCMV drove the expression of the chimeric activator carrying the strong VP64 AD. The two promoters caused a statistically identical basal fluorescence (ns: p-value = 0.076; two-tailed Welch’s t-test—in red in every figure). DEG1t_pCYC1noTATA had a greater detectability than pCMV (3.91 and 15.62 nM β-estradiol, respectively) and returned the highest fluorescence intensity. (**C**) Biosensors 3. The presence of the full pCYC1 5′UTR enhanced the maximal fluorescence level with respect to biosensors 1 and 2 without any relevant increment in the basal fluorescence. The fluorescence values of the three versions of biosensor 3 at 500, 1000, and 2000 nM β-estradiol did not show any statistically significant difference (ns: one-way ANOVA; p-value = 0.3224, 0.4226, and 0.2130, respectively).

These initial configurations highlighted some interesting features (see Fig. 3A). First, pGPD produced too many chimeric activators such that a fraction of them probably escaped the interaction with Hsp90 in the cytoplasm and diffused to the nucleus in the absence of β-estradiol. This caused a very high basal fluorescence (1237.13 A.U.) when the activator carried VP64 (byMM109). Both VP16 (byMM335) and mDR521-805 (byMM125) provoked a moderate basal fluorescence (372.19 and 228.32 A.U., respectively), whereas an extremely low value (1.58 A.U.) was due to B42 (byMM187). VP64 determined both the lowest tolerance—since fluorescence dropped after reaching its peak (3142.85 A.U.) at 7.81 nM β-estradiol—and the shortest detection range (from 3.91 to 7.81 nM). byMM335 (VP16) worked from 1.95 to 62.5 nM β-estradiol and expressed its highest fluorescence (2797.11) at 15.62 nM of the inducer. byMM125 (mDR521-805) further broadened the detection range (1.95 to 250 nM β-estradiol) and returned the highest fluorescence (3088.08 A.U.) at 125 nM β-estradiol. It should be noted that there was no statistically significant difference among the maximal fluorescence levels of these three biosensors hosting a strong AD (one-way ANOVA, p-value = 0.4843). Furthermore, the mean value of the highest fluorescence here measured corresponded to only 17.09% of that constitutively expressed by pGPD (18,318.37 ± 1318.32 A.U.—see Table S1). As for B42, all fluorescence values between 0 and 15.62 nM β-estradiol were lower than the background fluorescence. Hence its detection range went from 31.25 to 2000 nM β-estradiol, i.e., the maximal concentration used in this work.

This first biosensors pointed out a general pattern: strong ADs cause high D (i.e., they permit to detect small concentrations of β-estradiol) and low tolerance. In contrast, weak ADs determine a lower D and a higher tolerance.

Overall, none of the four biosensors was able to detect 0.98 nM β-estradiol. Moreover, as already mentioned above, the highest fluorescence levels were rather modest.

### Using weaker promoters in the receptor to decrease basal fluorescence and avoid toxicity effects

Biosensor 2 design: constitutive promoter-LexA-HBD(hER)-VP64 and 7×lex2Op(2, 37-TATA_-52_)-truncated_pCYC1min (see Fig. S7).

In biosensors 1, pGPD appeared to be too strong if the chimeric activator carried a potent AD. High basal fluorescence and low tolerance were the drawbacks of this configuration. Thus, we modified the receptor part of biosensors 1 by employing two weaker promoters to express the chimeric activator (still carrying VP64). We chose the synthetic promoter DEG1t_pCYC1noTATA (19.75% as strong as pGPD^25^) and the viral *CMV* promoter (pCMV, 4.74% as pGPD^26^—see Table S1). With respect to pGPD, the expression of the synthetic activator decreased drastically (see Fig. S2). Both DEG1t_pCYC1noTATA and pCMV reduced the basal fluorescence due to VP64 (458 ± 38.97 A.U. and 326 ± 76.01 A.U., respectively). Moreover, they did not show any toxicity effects up to the maximal concentration of β-estradiol. DEG1t_pCYC1noTATA turned out to be a better choice than pCMV since it guaranteed a larger detection interval (3.91–2000 nM β-estradiol) and reached a maximal fluorescence level (3242.78 A.U.) comparable to that of biosensors 1 (p-value = 0.5650; two-tailed Welch’s t-test), though at a much higher concentration of β-estradiol (2000 nM—see Fig. 3B).

### Enhancing fluorescence expression by restoring the full 5′UTR of the target promoter in the reporter

Biosensor 3 design: pGPD-LexA-HBD(hER)-B42 and 8×lex2Op(2, variable distance-TATA_-52_)-truncated_pCYC1min/core (see Fig. S8).

In order to increase the maximal fluorescence signal, we restored, in the reporter part, the full 5′UTR of the *CYC1* promoter, which permitted us to design three new synthetic promoters where transcription was activated by LexA-HBD(hER)-AD. They contained a cassette of eight lex2Op (separated by 2 nt—GT) that was followed by either pCYC1min (in two cases) or pCYC1core (as defined in Fig. 2). Each promoter was also characterized by a different distance between the 8×lex2Op cassette and TATA_-52_. On the receptor side, we chose the combination of pGPD and B42 AD in order to avoid toxicity. The three biosensors behaved quite similarly and pointed out that the full 5′UTR is necessary to achieve high fluorescence levels upon transcription activation (see Fig. 3C). Only one biosensor (byMM1376) had a basal fluorescence higher than the background signal. The maximal fluorescence outputs did not show any statistically significant difference among the three biosensors (see Fig. 3C caption). However, pCYC1core (byMM1378), which also contains TATA_-106_, reached the fluorescence peak (9312.6 A.U) at a lower β-estradiol concentration (500 nM) than that of the two pCYC1min-based biosensors (byMM1377: 1000 nM; byMM1376: 2000 nM). It should also be noted that the mean maximal fluorescence of byMM1378 turned out to be 5.55-fold higher than that of biosensor 1 byMM187 (our main reference, here) and 2.87-fold greater than that of biosensor 2 byMM198, corresponding to 50.64% of that of pGPD. Furthermore, both byMM1376 and byMM1378 showed a higher detectivity than that of byMM187 (31.25 nM), i.e., D = 7.81 nM and 15.62 nM, respectively.

### Enhancing fluorescence expression by varying the distance between a single lex2Op and TATA_-52_ in the promoter of the reporter

Biosensor 4 design: DEG1t_pCYC1noTATA-LexA-HBD(hER)-VP64 and lex2Op(variable distance-TATA_-52_)_pCYC1core (see Fig. S9).

From biosensors 3, it was apparent that we needed to keep the full *CYC1* 5′UTR in order to increase the maximal fluorescence level. Moreover, pCYC1core provided a higher detectivity than pCYC1min. In order to further improve the performance of our biosensors, we started making changes on the location of lex2Op.

Initially, we replaced the operator cassette with a single lex2Op inside pCYC1core and we varied its distance from TATA_-52_. In the receptor part, we adopted the configuration DEG1t_pCYC1noTATA-VP64, i.e., the most promising from biosensors 2.

**Figure 4:**
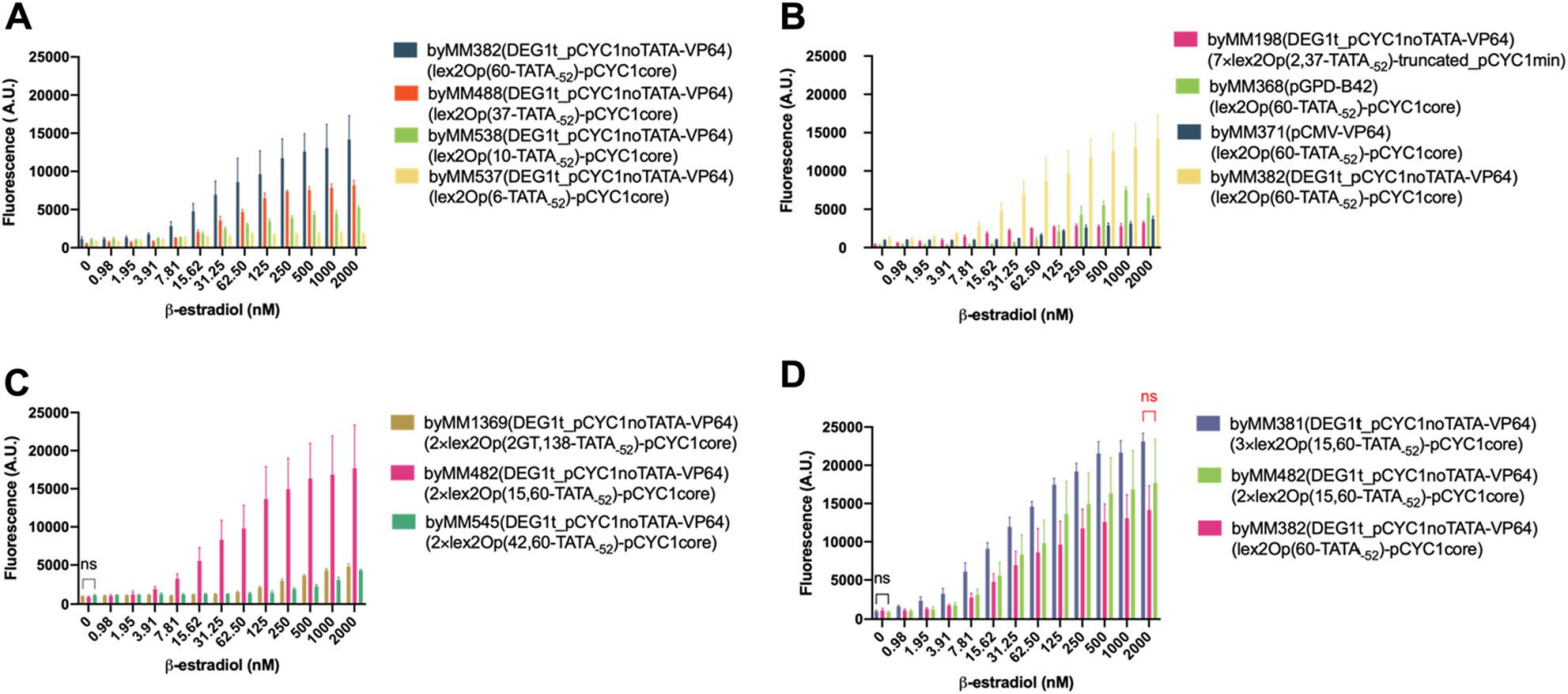
Response of biosensors 4–7 to increasing concentrations of β-estradiol. (**A**) Biosensors 4. A high distance—60 nt—between a single lex2Op and TATA_-52_ favors fluorescence expression. VP64 is probably the main responsible for the high basal fluorescence that went from 549.32 (byMM488) to 1142.28 A. U. (byMM382). (**B**) Biosensors 5. The receptor configurations pGPD-B42 (byMM368) and pCMV-VP64 (byMM371) fail to approach DEG1t_pCYC1noTATA-VP64 in the activation of a single lex2Op (byMM382). byMM371 reminds, at high concentrations of β-estradiol, byMM198. The latter, however, has a much higher detectivity (D = 3.91 nM instead of 125 nM). (**C**) Biosensors 6. A 15-nt distance between two adjacent lex2Op maximizes the output signal and guarantees a reasonably high detectivity. The three configurations have in common (no statistically significant difference—ns: p-value: 0.0810; one-way ANOVA) a rather high basal fluorescence, from 912.04 (byMM482) to 1136 A.U. (byMM545). (**D**) Biosensor 7. byMM381 fluorescence level is plotted in comparison with those of byMM482 (2×lex2Op) and byMM382 (lex2Op). The maximal mean fluorescence value of byMM381 is fairly higher than those of the other two biosensors (all at 2000 nM β-estradiol). However, there is no statistically significant difference between byMM381 and byMM482 maximal fluorescence level (ns: p-value = 0.0278; two-sided Welch’s t-test) due to the high standard deviation associated with the mean value of byMM482 fluorescence. No statistically significant difference is present among the three basal fluorescence either (ns: p-value: 0.4415; one-way ANOVA). byMM381 detectivity (1.95 nM β-estradiol) is, in contrast, much higher than that of the other two biosensors (7.81 nM β-estradiol). We tested similar cassettes of lex2Op also in front of the truncated_pCYC1core promoter (biosensors 10, see Fig. S15). The results are shown in Fig. S3.

In general, in order to have activation of transcription, the operator, where the activator binds, shall be upstream of, but not too close to, the TATA box. Otherwise, the activator, once bound to the DNA, will prevent RNA polymerase II from getting access to the promoter^27^. The lowest lex2Op-TATA_-52_ distance that we tested corresponded to 6 nt, which led to both a poor maximal fluorescence value (1933.79 A.U.) and a low detectivity (D = 62.5 nM β-estradiol). By increasing the distance between lex2Op and TATA_-52_, the maximal fluorescence grew steadily: 5222.21 A.U. (10 nt); 8186.36 A.U. (37 nt); and 14,170.70 A.U. (60 nt—byMM382—corresponding to 77.05% of pGPD fluorescence; see Fig. 4A).

It should be noted that, when lex2Op is 60 nt far from TATA_-52_, it is also 6 nt upstream of TATA_-106_, which could give a (probably modest) contribution to the final fluorescence level. Interestingly, the detectivity improved with the distance as well. However, the best value (D = 7.81 nM β-estradiol) was reached already at 37 nt. The basal fluorescence was, in general, rather high. However, no specific relation with the lex2Op-TATA_-52_ distance could be found.

### Confirming the best configuration for the receptor by testing two more constitutive promoters

Biosensor 5 design: constitutive promoter-LexA-HBD(hER)-AD and lex2Op(60-TATA_-52_)_ pCYC1core (see Fig. S10).

After selecting lex2Op(60-TATA_-52_)_pCYC1core as a reporter part, we constructed two new circuits where the receptor TU consisted of configurations tried in previous experiments, namely pGPD-B42 and pCMV-VP64 (i.e., a strong and a week element together). As reported in Fig. 4B, byMM368 (pGPD-B42) outperformed byMM371 (pCMV-VP64) in terms of both maximal and basal fluorescence (numerical values for all biosensor parameters are given in Table 1). However, both designs did not approach the performance of byMM382 and were not considered in further analysis.

**Table 1:** Overview of the biosensors’ performance.

Strain	Receptor	Reporter	Max fluor. (A.U.)	Conc. (nM)	Perc. pGPD (%)	Basal fluor. (A.U.)	Detectivity (nM)	Tolerance (nM)	Bio number
byMM109	pGPD-VP64	7×lex2Op(2, 37-TATA-52)-truncated_pCYC1min	3142.85	7.81	17.09	1237.13	3.91	7.81	1
byMM125	pGPD-mDR521-805	7×lex2Op(2, 37-TATA-52)-truncated_pCYC1min	3088.08	125	16.79	228.32	1.95	250	1
byMM187	pGPD-B42	7×lex2Op(2, 37-TATA-52)-truncated_pCYC1min	1676.16	500	9.11	1.58	31.25	2000	1
byMM335	pGPD-VP16	7×lex2Op(2, 37-TATA-52)-truncated_pCYC1min	2797.11	15.62	15.21	372.19	1.95	62.5	1
byMM198	DEG1t_pCYC1noTATA-VP64	7×lex2Op(2, 37-TATA-52)-truncated_pCYC1min	3242.78	2000	17.63	458	3.91	2000	2
byMM372	pCMV-VP64	7×lex2Op(2, 37-TATA-52)-truncated_pCYC1min	2077.96	1000	11.30	326.47	15.62	2000	2
byMM1376	pGPD-B42	8×lex2Op(2, 61-TATA-52)-pCYC1min	9231.65	2000	50.20	192.04	15.62	2000	3
byMM1377	pGPD-B42	8×lex2Op(2, 37-TATA-52)-pCYC1min	8724.82	1000	47.44	9.81	31.25	2000	3
byMM1378	pGPD-B42	8×lex2Op(2, 138-TATA-52)-pCYC1core	9312.6	500	50.64	42	15.62	2000	3
byMM382	DEG1t_pCYC1noTATA-VP64	lex2Op(60-TATA)-pCYC1core	14,170.70	2000	77.05	1142.28	7.81	2000	4
byMM488	DEG1t_pCYC1noTATA-VP64	lex2Op(37-TATA)-pCYC1core	8186.36	2000	44.51	549.32	7.81	2000	4
byMM538	DEG1t_pCYC1noTATA-VP64	lex2Op(10-TATA)-pCYC1core	5222.21	2000	28.40	1087.48	31.25	2000	4
byMM537	DEG1t_pCYC1noTATA-VP64	lex2Op(6-TATA)-pCYC1core	1933.79	1000	10.52	839.91	62.5	2000	4
byMM368	pGPD-B42	lex2Op(60-TATA)-pCYC1core	7499.76	1000	40.78	382.48	62.5	2000	5
byMM371	pCMV-VP64	lex2Op(60-TATA)-pCYC1core	3729.25	2000	20.28	953.32	125	2000	5
byMM1369	DEG1t_pCYC1noTATA-VP64	2×lex2Op(2GT,138-TATA)_pCYC1core	4801.21	2000	26.11	1029.71	250	2000	6
byMM482	DEG1t_pCYC1noTATA-VP64	2×lex2Op(15,60-TATA)_pCYC1core	17,707.27	2000	96.28	912.04	7.81	2000	6
byMM545	DEG1t_pCYC1noTATA-VP64	2×lex2Op(42,60-TATA)_pCYC1core	4286.55	2000	23.31	1136.85	1000	2000	6
byMM381	DEG1t_pCYC1noTATA-VP64	3×lex2Op(15,60-TATA)_pCYC1core	23,114.96	2000	125.69	1010.08	1.95	2000	7
byMM1396	DEG1t_pCYC1noTATA-VP64	3×lex2Op(2GT,138-TATA)_pCYC1core	9919.92	250	53.94	1663.96	7.81	2000	8
byMM496	DEG1t_pCYC1noTATA-VP64	3×lex2Op(6,60-TATA)_pCYC1core	25,378.69	2000	138.00	1074.93	3.91	2000	8
byMM487	DEG1t_pCYC1noTATA-VP64	3×lex2Op(21,60-TATA)_pCYC1core	23,895.19	2000	129.93	939.43	1.95	2000	8
byMM1397	DEG1t_pCYC1noTATA-VP64	3×lex2Op(42,60-TATA)_pCYC1core	18,083.71	125	98.33	3934.32	7.81	2000	8
byMM539	DEG1t_pCYC1noTATA-VP64	3×lex2Op(2,60-TATA)_pCYC1core	22,363.97	2000	121.61	595.63	1.95	2000	8
byMM498	DEG1t_pCYC1noTATA-VP64	3×lex2Op(9,60-TATA)_pCYC1core	22,222.01	250	120.83	2292.58	1.95	2000	8
byMM367	pGPD-B42	3×lex2Op(15,60-TATA)_pCYC1core	22,467.62	2000	122.17	11.32	62.5	2000	9
byMM369	pCMV-VP64	3×lex2Op(15,60-TATA)_pCYC1core	12,117.93	2000	65.89	734.59	3.91	2000	9
byMM1446	DEG1t_pCYC1noTATA-VP64	lex2Op(60-TATA)_truncated_pCYC1core	455.58	1000	2.48	35.01	1.95	2000	S10
byMM1448	DEG1t_pCYC1noTATA-VP64	2×lex2Op(15,60-TATA)_truncated_pCYC1core	2996.48	1000	16.29	98.88	3.91	2000	S10

### Increasing fluorescence expression by engineering the operator cassette of the promoter in the reporter. Varying the distance between two lex2Op

Biosensor 6 design: DEG1t_pCYC1noTATA-LexA-HBD(hER)-VP64 and 2×lex2Op(variable distance, 60/138-TATA_-52_)_ pCYC1core (see Fig. S11).

A way to further increase transcriptional activation demands to place several copies of the same operator upstream of the promoter TATA box^19,23,24^. Moreover, the distance between adjacent promoters can be used to modulate the transcription initiation rate^28^. We constructed three more promoters for the reporter TU. They were characterized by the presence of two lex2Op (2×lex2Op) far upstream of TATA_-52_ (i.e., 60 or 138 nt) of pCYC1core. They were spaced with 2, 15, and 42 nt. The receptor made use of the DEG1t_pCYC1noTATA-VP64 configuration. As shown in Fig. 4C and Table 1, 15 nt turned out to be an adequate distance between the two lex2Op (byMM482), whereas both 2 and 42 nt performed poorly, especially concerning the detectivity. Remarkably, the maximal fluorescence of byMM482 reached 17,707.27 A.U., i.e., 96.28% of that of pGPD. The detection range was also reasonably wide, from 7.81 up to 2000 nM β-estradiol.

### Adding a third lex2Op to the activated promoter

Biosensor 7 design: DEG1t_pCYC1noTATA-LexA-HBD(hER)-VP64 and 3×lex2Op(15, 60-TATA_-52_)_ pCYC1core (see Fig. S12).

By merging all our previous results, we were able to construct a highly performant biosensor just by adding a third lex2Op 15 nt upstream of the 2×lex2Op(15,60-TATA_-52_)_pCYC1core employed in byMM482. The new device returned a maximal fluorescence (23,114.96 A.U.) even 1.26-fold higher than that of pGPD and the overall broadest detection range (from 1.95 to 2000 nM β-estradiol). Only the basal fluorescence showed a rather high value (1010.08 A.U.), comparable to those of the circuits with only one or two lex2Op (see Fig. 4D).

### Varying the distance between adjacent lex2Op in the 3×lex2Op configuration of the activated promoter

Biosensor 8 design: DEG1t_pCYC1noTATA-LexA-HBD(hER)-VP64 and 3×lex2Op(variable distance, 60-TATA_-52_)_ pCYC1core (see Fig. S13).

Even though biosensor 7 turned out to work remarkably well, it was built on an approximative analysis of biosensors 6. We considered only three possible distances between the two lex2Op (2, 15, and 42 nt). Moreover, byMM1369 (2 nt) had a much longer distance from TATA_-52_ (138 nt) than the other two biosensors 6 (60 nt). Since 3×lex2Op permitted to achieve both a fluorescence level greater than that of pGPD and a high detectability, we decided to investigate if the performance of biosensor 7 could be further improved by varying the separation between two contiguous lex2Op. Thus, we built five more 3×lex2Op where two consecutive lex2Op were separated by 2, 6, 9, 21, and 42 nt with the overall cassette placed 60 nt upstream of TATA_-52_. Furthermore, for a comparison with biosensor 6 byMM1369, we constructed another biosensor where the three operators were separated by GT and the whole 3×lex2Op cassette lied 138 nt upstream of TATA_-52_ (byMM1396, we will refer to it as 2-GT).

As for the basal fluorescence, there was no statistically significant difference among the 6, 15, and 21 nt configuration (ns: p-value = 0.4907, one-way ANOVA—fluorescence oscillated around 1000 A.U.), whereas 9 nt expressed over 2200 A.U. of fluorescence in the absence of β-estradiol. Forty-two and two nt returned the highest (3934.32 A.U.) and the lowest (595.63 A.U.) value, respectively, whereas 2-GT expressed 1663.96 A.U. Compared to the 2×lex2Op configuration, the basal fluorescence increased in a statistically significant way only for 2-GT and 42 nt (p-value < 0.0001; two-sided Welch’s t-test), whereas the increment in 15 nt was not significant (p-value = 0.4507; two-sided Welch’s t-test—see Table 1).

**Figure 5:**
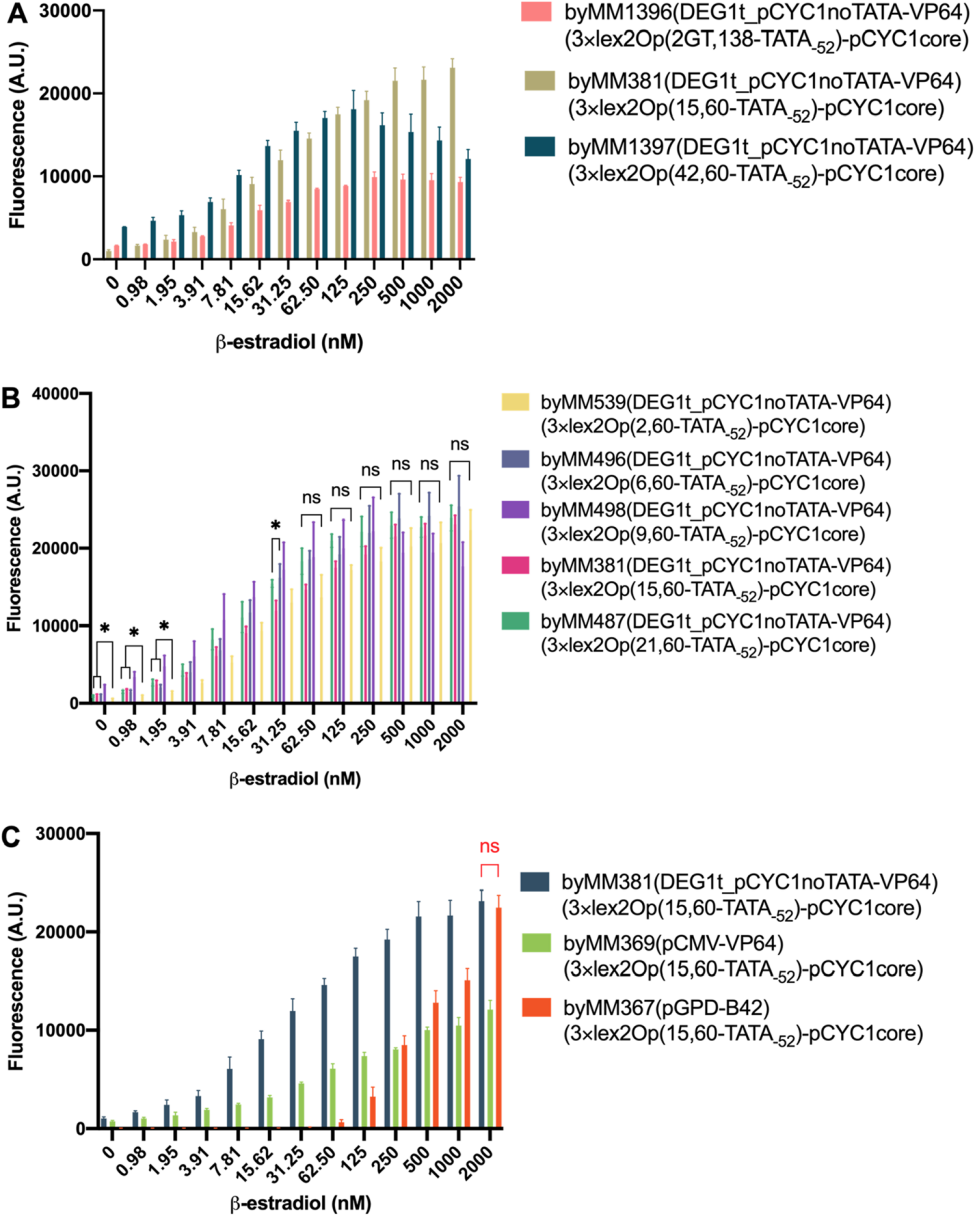
Response of biosensors 8–9 to increasing concentrations of β-estradiol. (**A**) Comparison of the performance of biosensor 7 (byMM381) and biosensors 8 (byMM1396, byMM1397). The three of them were built by adding one lex2Op to a different biosensor 6 configuration. (**B**) Comparative response of biosensor 7 and four variants. The biosensors differ from each other only for the distance between two adjacent lex2Op (2, 6, 9, 15, and 21 nt). In particular, four configurations (2, 6, 15, and 21 nt) appear highly effective and almost undistinguishable from each other. Details about the statistical analysis are given in the main text. (**C**) Biosensors 9. byMM369 is completely outperformed by byMM381, whereas byMM367 share, with byMM381, the maximal fluorescence value (ns: p-value = 0.5360; two-tailed Welch’s t-test) and has a much lower basal fluorescence. The circuit in byMM367 is not optimal as a biosensor but it is extremely valuable to activate and enhance gene expression at 2000 nM β-estradiol, with basically no leakage when the chemical is absent.

The maximal fluorescence was reached at 2000 nM β-estradiol and overcame 120% of that of pGPD in the majority of the biosensor configurations. Exceptions were 2-GT (53.94%, at 250 nM) and 42 nt (98.33% at 125 nM). Higher basal fluorescence and lower maximal output made the titration curves of 2-GT and 42 nt (byMM1397) clearly different from that of biosensor 7 (byMM381), as apparent in Fig. 5A.

By looking at the three biosensors containing 2×lex2Op, the addition of the third operator was beneficial both for the maximal fluorescence and the detectivity: 2-GT maximal fluorescence showed a 2.07-fold increase and D went from 250 to 7.81 nM β-estradiol. The improvements on 42 nt performance were even more evident: a 4.22-fold increment in the maximal fluorescence and D from 1000 to 7.81 nM β-estradiol. Less apparent were the improvements on the already highly efficient 15 nt: 1.30-fold augment in the maximal fluorescence and D from 7.81 to 1.95 nM β-estradiol. Therefore, despite causing an increase in basal fluorescence, a third lex2Op provided an overall advancement in the biosensors’ performance (see Fig. 5A).

A striking result from our analysis was that by varying the distance between adjacent lex2Op from 2 to 21 nt, no big change arose in the circuit behavior. More precisely, we took into account five distances: 2, 6, 9, 15, and 21 nt; 6, 15, and 21 nt showed statistically significant difference only at 31.25 nM β-estradiol (*: p-value = 0.0153; one-way ANOVA), otherwise their titration curves were basically equivalent. Two nt differed from the previous three configuration in four cases only: 0, 0.98, 1.95 and 31.25 nM β-estradiol. Nine nt reached a remarkable maximal output, corresponding to 120.83% of pGPD fluorescence, at only 250 nM β-estradiol. It should be noted, though, that the high error associated with 9 nt measurements made the fluorescence levels between 31.25 and 2000 nM β-estradiol undistinguishable in statistical terms (p-value = 0.6899; one-way ANOVA). Finally, there was no significant difference among the five biosensors in the range 62.5–2000 nM (more than one third of the whole measurements), i.e., at the concentrations of β-estradiol where the highest fluorescence values were reported. Thus, the maximal fluorescence values of the five circuits turned out not to be significantly different in statistical terms (p-value = 0.6974; one-way ANOVA). Furthermore, each configuration showed high detectability: 1.95 (2, 9, 15, and 21 nt) and 3.91 (6 nt) nM β-estradiol (see Fig. 5B and Table 1).

Taken together, we realized a highly efficient β-estradiol biosensor via the combination of a receptor characterized by the synthetic promoter DEG1t_pCYC1noTATA and the VP64 AD together with a reporter containing a 3×lex2Op cassette 60 nt upstream of TATA_-52_ of the *CYC1* core promoter (which retains its full natural 5′UTR). When the distance between adjacent lex2Op was between 2 and 21 nt, every biosensor was characterized by a very broad detection range (from, at least, 3.91 to 2000 nM β-estradiol no toxicity effects were present) and a mean maximal fluorescence value at least 1.20-fold higher than that produced by the strong *GPD* promoter. Therefore, our device can also be adopted to upregulate gene expression.

### Turning the biosensor into a switch by changing the receptor configuration

Biosensor 9 design: constitutive promoter-LexA-HBD(hER)-AD and 3×lex2Op(15, 60-TATA_-52_)_pCYC1core (see Fig. S14).

After finding five efficient designs for the promoter in the reporter part, we chose the configuration in biosensor 7—3×lex2Op(15,60-TATA_-52_)_pCYC1core—and checked if it could lead to the construction of more performant biosensors where pGPD-B42 and pCMV-VP64 took in the receptor part the place of DEG1t_pCYC1noTATA-VP64. Previously, in biosensors 5, both configurations did not work very well by acting on a single lex2Op placed 60 nt upstream of TATA_-52_.

pCMV-VP64 together with an optimized 3×lex2Op cassette (byMM369) increased both the maximal fluorescence value (from 26.32 to 65.89% of pGPD one) and the detectivity (from 125 to 3.91 nM β-estradiol) of byMM371. However, it remained overall far from the performance of most of the biosensors 8. The results obtained with pGPD-B42 appeared more interesting. The new biosensor byMM367 had an extremely low basal fluorescence (11.32 A.U.) but needed 31.25 nM β-estradiol to overcome the background fluorescence, which limited its detection range. At 250 nM β-estradiol, byMM367 fluorescence level was comparable to that of byMM369, thus clearly lower (44.15%) that than of byMM381 but, then, increased sharply and reached a remarkable maximal value (122.17% of pGPD one) at 2000 nM β-estradiol (see Fig. 5C). Overall, the circuit based on pGPD-B42 guarantees a strong gene expression at 2000 nM concentration of β-estradiol and almost no leakage when the hormone is absent.

It should be noted, though, that higher concentrations of β-estradiol (from 4000 nM up to 16,000 nM) spoil the working of byMM367 with the appearance of toxicity, whereas they have no relevant effects on biosensors containing the configuration DEG1t_pCYC1noTATA-VP64 in the receptor and one up to three lex2Op in the reporter (byMM382, byMM482, and byMM381—see Figs. S4, S5).

On the whole, byMM367 shall be used as a switch (rather than a biosensor) between 0 and 2000 nM β-estradiol to turn OFF and ON effectively—without any problems for the cells—the expression of a target gene.

## Conclusions

Chimeric proteins made of domains that are not expressed in yeast are likely to be orthogonal to the yeast genome and, therefore, represent good candidates for wiring the TUs present in a synthetic circuit^29^. Bi- or tripartite fusion proteins hosting the docking site for a small molecule permit, furthermore, to control the circuit working from the outside. HBD(hER) has allowed the construction of several chimeric transcription factors responding to β-estradiol, a hormone well-tolerated by the yeast *S. cerevisiae*. In this work, we focused on a set of synthetic activators where the bacterial LexA had the function of DBD and it was fused to HBD(hER) and an AD of viral, bacterial, or even mammalian origin. As previously mentioned, this chimeric protein is not new and several variants—together with their target promoters built ad hoc—have been adopted^16,18,30–33^. We constructed ten different types of β-estradiol biosensors in order to find an optimal configuration that combined a very high fluorescence expression (higher than that reached by the strong *GPD* promoter) and a broad detection range. To this aim, we had to assess different configurations of the receptor part (the constitutive promoter and the AD) and engineer target promoters in the reporter part. In our analysis, the best promoter, in the reporter, was made by the core *CYC1* sequence (which keeps the three TATA boxes and the full 5′UTR) preceded by three lex2Op operators. Adjacent lex2Op shall be separated by 2 up to 21 nt and the 3×lex2Op cassette is placed 60 nt upstream of TATA_-52_. On the receptor side, we found two useful configurations. The best biosensor configuration demands to fuse LexA-HBD(hER) to the strong VP64 AD and express it under the synthetic constitutive DEG1t_pCYC1noTATA promoter. In this way, the maximal fluorescence level overcomes that of the *GPD* promoter (1.38-fold higher when 6 nt separate two lex2Op) and the detection range goes from 1.95 to 2000 nM (2, 9, 15, and 21 nt). In contrast, by replacing VP64 with B42 and DEG1t_pCYC1noTATA with pGPD, we obtain a perfect switch that expresses, at 2000 nM β-estradiol, more fluorescence than pGPD and shows a negligible leakage in the absence of β-estradiol.

By comparing our results to those by Ottoz et al*.*^17^, our main reference, we can see that our promoter leads to a stronger gene expression with a lower number of lex2Op (three instead of eight). Hence, we might achieve a further enhancement in gene expression by adding more lex2Op. The core promoter in^17^ was a minimal version of pCYC1 that retained a single TATA box starting at position -46 with respect to the TSS (not at -52 because TATA_-22_ was deleted) and had a long 5′UTR (77 nt of which the last 41 nt came from the plasmid MCS—multiple cloning sequence). The distance between two adjacent lex2Op was 6 nt (in the optimal range for high gene expression, according to our analysis), whereas the lex2Op cassette lied only 22 nt upstream of TATA_-46_, i.e., much less than in our target promoter. On the receptor side, Ottoz et al*.* obtained their best results by using B112 AD and the pACT1 promoter. When using VP16, they reported toxicity effects between 8 and 31 nM β-estradiol. Interestingly, our optimized biosensors did not show any toxicity despite the fact that VP64 was expressed under DEG1t_pCYC1noTATA that is 1.37-fold stronger than pACT1. This seems to point out that toxicity is not caused only by the kind and the amount of the ADs expressed in the cells. Strong ADs in high concentration are, indeed, supposed to prevent the synthesis of vital genes—and lead to cell death—by reducing the availability of RNA polymerase II molecules^20^. However, different chimeric activators that are made of dCas9:gRNA (guide RNA) or dCas12a:crRNA (CRISPR RNA) fused to VP64 or the stronger VPR have been expressed in *S. cerevisiae* under pGPD without inducing any toxicity effects^23,24,27^. Therefore, we think that we still have to fully understand what causes toxicity, in yeast cells, in the presence of β-estradiol and LexA-HBD(hER)-AD. Only this knowledge will permit us to include, in a safe and reliable way, this system inside more complex synthetic gene networks that respond to a combination of multiple inputs.

## Materials and methods

### Plasmid construction

The plasmids realized in this study (see Table S2) are based on the yeast-integrative shuttle vector pRSII405 (receptor part) and pRSII406 (reporter part). Both backbones are available at Addgene (number 35440 and 35442, respectively; a gift from Steven Haase^34^). Every plasmid, which hosted a different transcription unit, was assembled with the Gibson method^35^. To this aim, the integrative backbone was cut-open (5 µg in a 30 µl solution) with the restriction endonucleases Acc65I (NEB-R0599S) and SacI (NEB-R0156S) to remove the MCS. At the end of the digestion, the two enzymes were heat-inactivated (20 min. at 65 °C). Every biological part (promoters, coding regions, and terminators) was amplified from their original plasmid via touchdown PCR^36^ that was carried out by using Q5 Hot Start high-fidelity DNA polymerase (NEB-M0493S). Primers were designed to guarantee a 40-nt-overlap between adjacent DNA sequences (see Table S4). PCR products were eluted from the agarose gel by means of the AxiPrep DNA extraction kit (Axigen-AP-GX-250). Biological parts and the cut-open backbone were mixed in equimolar amount in a 5 µl solution, which was added later to 15 µl of Gibson master mixture solution (main components: T5 exonuclease (NEB-M0363), Phusion High-Fidelity DNA polymerase (Thermo Scientific-F530L), and Taq DNA ligase (NEB-M0208L)). The overall 20 µl solution was kept in a Thermal Cycler for 1 h at 50 °C. *Escherichia coli* competent cells (strains DH5α—Life Technology, 18263-012) were transformed with the plasmids that resulted from the Gibson assembly via a thermal shock (30 s at 42 °C)^36^. All plasmids were sequenced at Genewiz Inc., Suzhou (China), to check the correctness of the DNA sequences corresponding to the different TUs.

### Yeast strain construction

Our plasmids were integrated into the genome of the yeast *S. cerevisiae* strain FY1679-08A (MATa; ura3-52; leu2D1; trp1D63; his3D200; GAL2)—EUROSCARF 10000M, Johann Wolfgang Goethe University, Frankfurt, Germany. Genomic integration was carried out according to the lithium-acetate protocol, as described in^37^, and required to linearize 5 µg of plasmid DNA with either the enzyme StuI (NEB-R0187V), to cut inside the URA3 marker, or BstEII (NEB-R0162S), to cleave the LEU2 marker. Transformed cells were grown for two days at 30 °C on plates (2% glucose, 2% agar) containing a synthetic defined selective medium: SD-URA or SD-LEU. All yeast strains engineered in this work are listed in Table S3.

### RT-qPCR

RNA extraction and purification from yeast cells (strains: byMM357, byMM367, byMM369, and byMM381) were carried via the YeaStar RNA kit (Zymo Research-R1002). cDNA was synthesized via the HiFiScript cDNA Synthesis Kit (CWBIO-CW2569M). The primers used to amplify a portion of LexA, Hsp90, and the reference ACT1 transcript are reported in Table S5. A qPCR solution had a 10 μL volume divided in: 5 μL 2xSYBR qPCR Mix (SparkJade AH0104); 0.2 μL 10 μM forward and reverse primers; 0.2 μL ROX(II); cDNA (from 20 to 50 ng); and RNase-free water. On a Roche LightCycler96 machine, we run the program: (1) hold stage: 2 min at 50 °C followed by 10 min at 95 °C; (2) PCR stage: 15 s at 95 °C, followed by 34 s at 55 °C. The PCR stage was cycled 45 times. Each sample was present in three replicates. Relative mean mRNA expression levels were calculated via the Pfaffl formula^38^. The standard deviation was determined through the error propagation formula.

### Growth curve

Yeast strains (byMM2 and byMM367) were grown in complete synthetic defined medium (SDC) at 30 °C and 240 RPM for 14 h. Cell concentration (OD600) was then measured such that cell solutions could be diluted to OD600 ~ 0.2 in 30 mL SDC. Yeast cells were grown for 20 h, and OD600 was measured every 2 h with an Eppendorf BioPhotometer device.

### Flow cytometry

Yeast cells were grown, first, for 14 h at 30 °C and 240 RPM in SDC. Cells were then 1:100 diluted in 2 ml SDC supplemented with varying concentrations of β-estradiol (Sigma-Aldrich—E8875; 10 mM stock solution in ethanol). They grew for 20 h at the same conditions described above. Before the FACS experiment, cells were 1:20 diluted (in SDC). To measure fluorescence intensity, we used a BD FACSVerse (blue laser 488 nm, emission filter 527/32). The FACS machine set-up was checked via the QC (quality check) program using fluorescent beads (BD FACS uiteTM CS&T Research Beads—17495). Every yeast strain was measured three times in different days. During each experiment, 10,000 events were collected.

### Data analysis

The flowcore R-Bioconductor package was used to analyze the data from BD FACSVerse^39^. The mean background fluorescence, measured on the chassis strain (byMM2) that does not contain any fluorescence source, was subtracted from the fluorescence intensity associated with each engineered strain.

## Supplementary Information

Supplementary Information.

## Data Availability

FACS data (fcs files) relating to the results in this work have been uploaded to FlowRepository (http://flowrepository.org) http://flowrepository.org/id/RvFrJsV75hjylPqY4nRk5jbOptOaYbA8XwUqGlylxSmEjZysGDGGHpXIfcHbnUpy.
