# Supplementary material for: Engineering a two-gene system to operate as a highly sensitive biosensor or a sharp switch upon induction with β-estradiol
Source: Sci Rep. 2022 Dec 16;12:21791. doi: 10.1038/s41598-022-26195-x (PMC9758199; doi:10.1038/s41598-022-26195-x)
Supplement: Supplementary file 1 — Supplementary Information. [file 41598_2022_26195_MOESM1_ESM.pdf]

Supplementary materials

Engineering a two-gene system to operate as a highly sensitive biosensor or a sharp switch upon induction with β-estradiol.

Tian Zhou[1,+], Zhiying Liang[2,+], and Mario Andrea Marchisio[1,*]

[1] School of Pharmaceutical Science and Technology, Tianjin University, 92 Weijin Road, 300072 Tianjin, China

[2] School of Life Science and Technology, Harbin Institute of Technology, 2 Yikuang Street, 150080 Harbin, China

[+] the authors contributed equally

*corresponding author. Email address: mario@tju.edu.cn or mamarchisio@yahoo.com

Figures

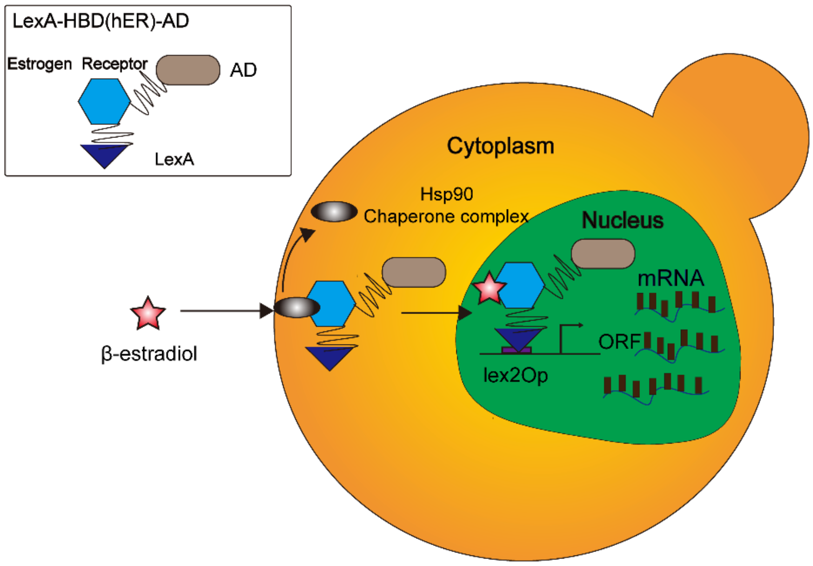

Figure S1. Action of β-estradiol on a chimeric activator containing the human estrogen receptor. In the absence of β-estradiol, Hsp90 binds the estrogen receptor and sequesters the chimeric activator in the cytoplasm. Upon induction with β-estradiol, the hormone docks to estrogen receptor hindering the binding of Hsp90. Thus, the chimeric activator can translocate into the nucleus, activate its target promoter, and lead to the expression of an output signal.

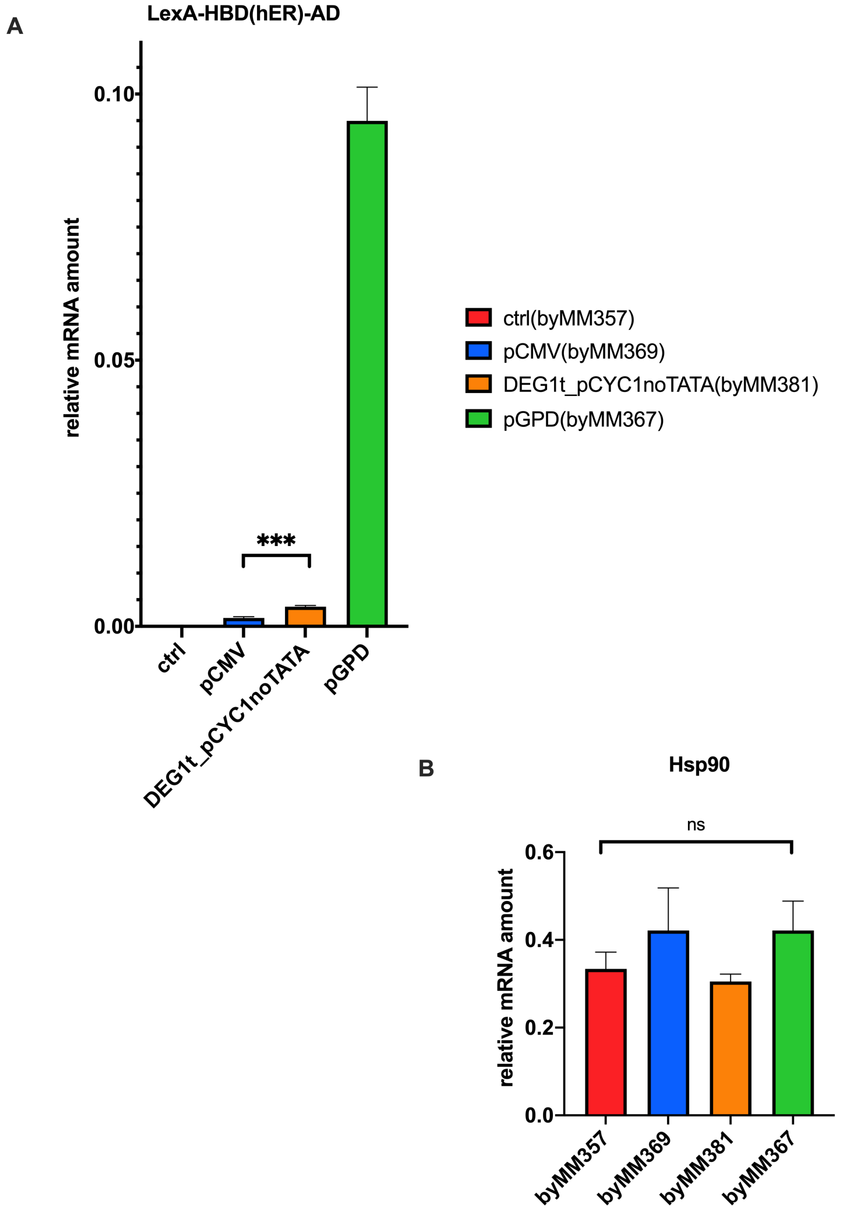

Figure S2. mRNA expression: the synthetic activator and the Hsp90 protein. A) The mRNA quantity associated with the synthetic activator and relative to that of the endogenous *ACT1* gene grows with the strength of the promoter in the receptor. The control (ctrl) strain, byMM357, contains only the reporter part of the β-estradiol biosensor (***: p-value < 0.001, two-sided Welch's t-test). B) The expression of Hsp90 is not affected by the presence of the synthetic activator in different amount (ns: no statistically significant difference; p-value = 0.1110, one-way ANOVA).

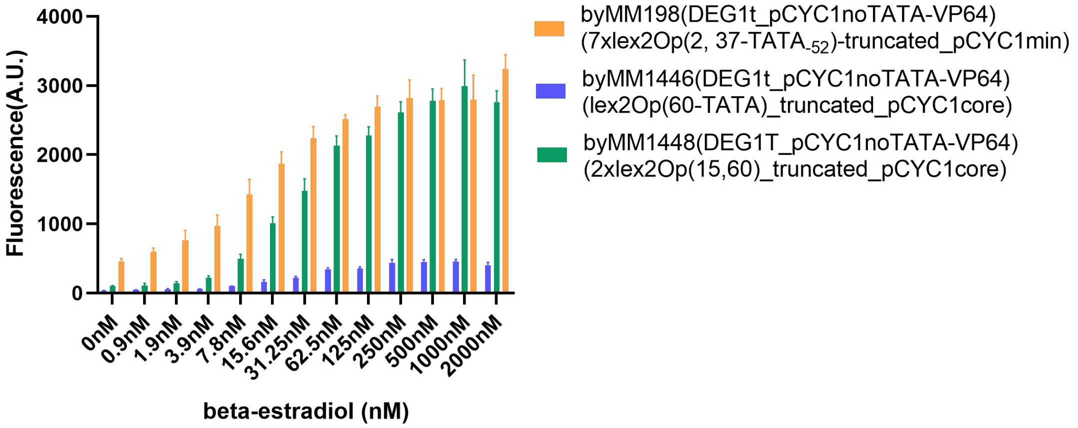

Figure S3. Response of biosensors 10 to increasing concentrations of β-estradiol. The weak truncated_pCYC1core is preceded by 1, or 2 lex2Op. Two adjacent lex2Op are 15-nt distant. A whole lex2Op cassette is 60 nt upstream of $TATA_{-52}$. Both maximal and basal fluorescence increased considerably from 1 to 2 lex2Op. The best detection range was 3.9÷2000 nM beta-estradiol (byMM1448). byMM198 (DEG1t_pCYC1noTATA-VP64 and 7×lex2Op(2, 37-$TATA_{-52}$)-truncated_pCYC1min) showed a titration curve similar to that of byMM1448, though with a higher basal fluorescence.

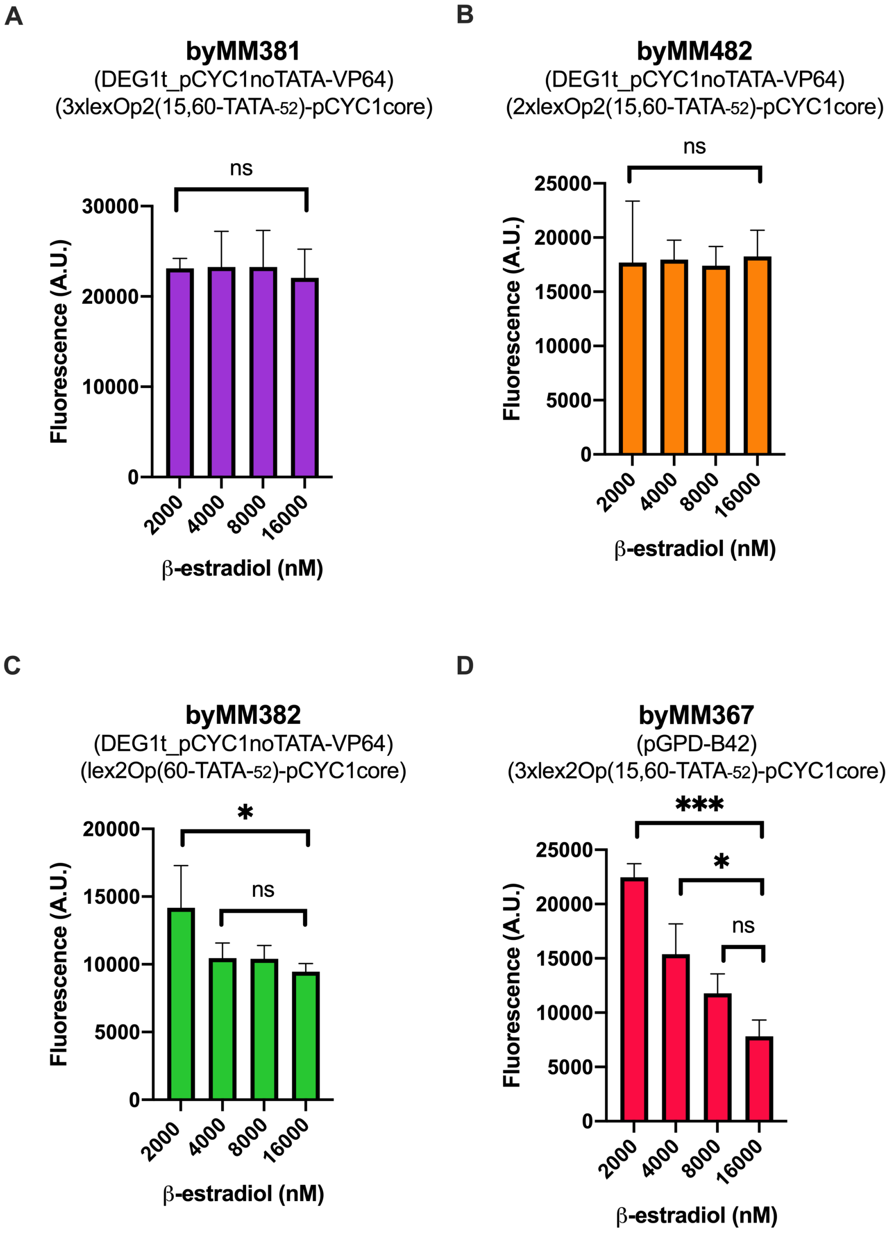

Figure S4. Response of four circuits to high concentrations of β-estradiol. A-C) Biosensors containing "DEG1t_pCYC1noTATA-VP64" in the receptor and a different number of lex2Op in the reporter. The fluorescence level between 2000 and 16000 nM β-estradiol stays constant in byMM381 (p-value = 0.9616, one-way ANOVA) and byMM482 (p-value = 0.9899, one-way ANOVA). A slight decrease is present in byMM382 between the fluorescence measured at 2000 nM β-estradiol and that at the other three concentrations (*: p-value <0.05; ns: p-value = 0.3927, one-way ANOVA). D) Switch circuit. Fluorescence drops constantly and considerably by increasing the concentration of β-estradiol, which signals possible toxicity in the cells (***: p-value < 0.001, *: p-value <0.05, one-way ANOVA; ns: p-value = 0.0876, two-sided Welch's t-test).

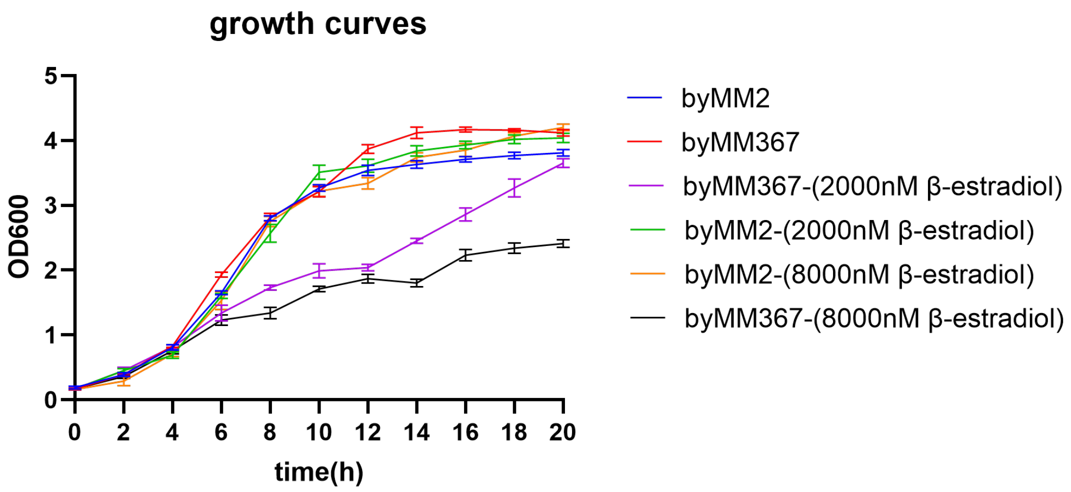

Figure S5. Growth curve of byMM2 (empty chassis) and byMM367 (switch circuit) at different concentration of β-estradiol. byMM2 growth is not affected by the presence of β-estradiol in the cell culture. In contrast, byMM367 appears to grow more slowly, especially at 8000 nM β-estradiol. This indicates that the circuit shall not be induced with more than 2000 nM β-estradiol.

Tables

Table S1. Fluorescence level reached by natural and synthetic yeast constitutive promoters. Each fluorescence value is the mean of three independent experiments.

Promoter	Mean fluorescence	Standard deviation of the mean
pGPD	18318.37	1318.32
DEG1t_pCYC1noTATA	3631.45	175.24
pCYC1min	3256.78	152.24
pACT1	2650.94	92.96
pCMV	872.15	14.52
trunc_pCYC1min	79.34	6.42

Table S2. Plasmids engineered in this project.

Plasmid name	Construct
pMM220	pRSII406-7×lex2Op(2, 37-TATA-52)-truncated_pCYC1min-yEG-CYC1t
pMM701	pRSII406-8×lex2Op(2, 61-TATA-52)-pCYC1min-yEGFP-CYC1t
pMM449	pRSII406-8×lex2Op(2, 37-TATA-52)-pCYC1min-yEGFP-CYC1t
pMM436	pRSII406-8×lex2Op(2,138-TATA-52)-pCYC1core-yEGFP-CYC1
pMM526	pRSII406-lex2Op(60-TATA)-pCYC1core-yEGFP-CYC1t
pMM661	pRSII406-lex2Op(37-TATA)-pCYC1core-yEGFP-CYC1t
pMM698	pRSII406-lex2Op(10-TATA)-pCYC1core-yEGFP-CYC1t
pMM697	pRSII406-lex2Op(6-TATA)-pCYC1core-yEGFP-CYC1t
pMM703	pRSII406-2×lex2Op(2GT,138-TATA)_pCYC1core-yEGFP-CYC1t
pMM659	pRSII406-2×lex2Op(15,60-TATA)_pCYC1core-yEGFP-CYC1t
pMM691	pRSII406-2×lex2Op(42,60-TATA)-pCYC1core-yEGFP-CYC1t
pMM527	pRSII406-3×lex2Op(15,60-TATA)_pCYC1core-yEGFP-CYC1t
pMM700	pRSII406-3×lex2Op(2GT,138-TATA)_pCYC1core-yEGFP-CYC1t
pMM655	pRSII406-3×lex2Op(6,60-TATA)_pCYC1core-yEGFP-CYC1t
pMM662	pRSII406-3×lex2Op(21,60-TATA)_pCYC1core-yEGFP-CYC1t
pMM696	pRSII406-3×lex2Op(2,60-TATA)_pCYC1core-yEGFP-CYC1t
pMM663	pRSII406-3×lex2Op(9,60-TATA)_pCYC1core-yEGFP-CYC1t
pMM527	pRSII406-3×lex2Op(15,60-TATA)_pCYC1core-yEGFP-CYC1t
pMM1374	pRSII406-lex2Op(60-TATA)_truncated_pCYC1core-yEGFP-CYC1t
pMM197	pRSII405-pGPD-lexA-HBD(hER)-VP64-CYC1t
pMM229	pRSII405-pGPD-lexA-HBD(hER)-mDR521_805-CYC1t

pMM403	pRSII405-pGPD-lexA-HBD(hER)-B42-CYC1t
pMM478	pRSII405-pGPD-lexA-HBD(hER)-VP16-CYC1t
pMM363	pRSII405-DEG1t-pCYC1noTATA-lexA-HBD(hER)-VP64-CYC1t
pMM555	pRSII405-pCMVC-lexA-HBD(hER)-VP64-CYC1t

Table S3. Synthetic yeast strains realized in this work.

Strains name	Genotype
byMM106	byMM2 pMM220 :: URA3
byMM1343	byMM2 pMM701 :: URA3
byMM1344	byMM2 pMM449 :: URA3
byMM1345	byMM2 pMM436 :: URA3
byMM362	byMM2 pMM526 :: URA3
byMM479	byMM2 pMM661 :: URA3
byMM532	byMM2 pMM698 :: URA3
byMM531	byMM2 pMM697 :: URA3
byMM1368	byMM2 pMM703 :: URA3
byMM477	byMM2 pMM659 :: URA3
byMM541	byMM2 pMM691 :: URA3
byMM357	byMM2 pMM527 :: URA3
byMM1382	byMM2 pMM700 :: URA3
byMM476	byMM2 pMM655 :: URA3
byMM480	byMM2 pMM662 :: URA3
byMM530	byMM2 pMM696 :: URA3
byMM501	byMM2 pMM663 :: URA3
byMM357	byMM2 pMM527 :: URA3
byMM1420	byMM2 pMM1374 ::URA3
byMM1421	byMM2 pMM1375 :: URA3
byMM1422	byMM2 pMM1376 :: URA3
byMM109	byMM2 pMM220 :: URA3 pMM197 :: LEU2
byMM125	byMM2 pMM220 :: URA3 pMM229 :: LEU2
byMM187	byMM2 pMM220 :: URA3 pMM403 :: LEU2
byMM335	byMM2 pMM220 :: URA3 pMM478 :: LEU2
byMM198	byMM2 pMM220 :: URA3 pMM363 :: LEU2
byMM372	byMM2 pMM220 :: URA3 pMM555 :: LEU2
byMM1376	byMM2 pMM701 :: URA3 pMM403 :: LEU2
byMM1377	byMM2 pMM449 :: URA3 pMM403 :: LEU2
byMM1378	byMM2 pMM436 :: URA3 pMM403 :: LEU2
byMM382	byMM2 pMM526 :: URA3 pMM363 :: LEU2
byMM488	byMM2 pMM661 :: URA3 pMM363 :: LEU2
byMM538	byMM2 pMM698 :: URA3 pMM363 :: LEU2
byMM537	byMM2 pMM697 :: URA3 pMM363 :: LEU2
byMM368	byMM2 pMM526 :: URA3 pMM403 :: LEU2
byMM371	byMM2 pMM526 :: URA3 pMM555 :: LEU2

byMM1369	byMM2 pMM703 :: URA3 pMM363 :: LEU2
byMM482	byMM2 pMM659 :: URA3 pMM363 :: LEU2
byMM545	byMM2 pMM691 :: URA3 pMM363 :: LEU2
byMM381	byMM2 pMM527 :: URA3 pMM363 :: LEU2
byMM1396	byMM2 pMM700 :: URA3 pMM363 :: LEU2
byMM496	byMM2 pMM655 :: URA3 pMM363 :: LEU2
byMM487	byMM2 pMM662 :: URA3 pMM363 :: LEU2
byMM1398	byMM2 pMM700 :: URA3 pMM363 :: LEU2
byMM539	byMM2 pMM696 :: URA3 pMM363 :: LEU2
byMM498	byMM2 pMM663 :: URA3 pMM363 :: LEU2
byMM367	byMM2 pMM527 :: URA3 pMM403 :: LEU2
byMM369	byMM2 pMM527 :: URA3 pMM555 :: LEU2
byMM1446	byMM2 pMM1374 :: URA3 pMM363 :: LEU2
byMM1448	byMM2 pMM1375 :: URA3 pMM363 :: LEU2

Table S4. Primers used in this work for plasmid assembly.

ID	Sequence	Dir.	Description
oMM34	GTGAGCGCGCGTAATACGACTCACTATAGGGCGAATTGGGTACCCAGTTCGAGTTTATC	fw	vector-pGPD
oMM37	CAATTAACCCTCACTAAAGGGAACAAAAGCTGGAGCTCGATAGCTTGCAAATTAAAGC	rev	CYC1t-vector
oMM48	CGCGCGTAATACGACTCACTATAGGGCGAATTGGGTACCAATAATATATAAACCTGTAT	fw	vector-DEG1t_pCYC1noTATA
oMM56	GCGCGCGTAATACGACTCACTATAGGGCGAATTGGGTACCCAGATCCGCCAGGCGTGT	fw	vector-pCYC1core
oMM57	GCGCGCGTAATACGACTCACTATAGGGCGAATTGGGTACCGCATGCATGTGCTCTGTA	fw	vector-pCYC1min
oMM58	ATACACACACTAAATTAATAATGTCTAAAGGTGAAGAATT	fw	pCYC1 (complete 5'UTR)-yEGFP
oMM59	AATTCTTCACCTTTAGACATTATTAATTTAGTGTGTGTAT	rev	pCYC1 (complete 5'UTR)-yEGFP
oMM60	TGGTATGGATGAATTGTACAAATAACATGTAATTAGTTATGTCACGCTTA	fw	yEGFP-CYC1t
oMM61	TAAGCGTGACATAACTAATTACATGTTATTTGTACAATTCATCCATACCA	rev	yEGFP-CYC1t
oMM74	AACACACATAAACAAACAAAATGAAAGCGTTAACGGCCAG	fw	pGPD-LexA
oMM75	CTGGCCGTTAACGCTTTCATTTTGTTTGTTTATGTGTGTT	rev	pGPD-LexA
oMM78	TTCGCAACGGCGACTGGCTGTCATCTGCTGGAGACATGAG	fw	LexA-HBD(hER)
oMM79	CTCATGTCTCCAGCAGATGACAGCCAGTCGCCGTTGCGAA	rev	LexA-HBD(hER)

oMM80	CTGCGGGCTCTACTTCATCGGACGCGCTGGACGATTTCGA	fw	HBD(hER)-VP64
oMM81	TCGAAATCGTCCAGCGCGTCCGATGAAGTAGAGCCCGCAG	rev	HBD(hER)-VP64
oMM87	TGTCTAAGGTTCAATCTTAACATGTAATTAGTTATGTCAC	fw	mDR521_805-CYC1t
oMM88	GTGACATAACTAATTACATGTTAAGATTGAACCTTAGACA	rev	mDR521_805-CYC1t
oMM339	GCGCGCGTAATACGACTCACTATAGGGCGAATTGGAGAGAAGACAAGAGC	fw	vector-8×lex2Op
oMM356	CTGCGGGCTCTACTTCATCGGAATTGTTCCCAGACAACAA	fw	HBD(hER)-mDR521_805
oMM357	TTGTTGTCTGGGAACAATTCCGATGAAGTAGAGCCCGCAG	rev	HBD(hER)-mDR521_805
oMM363	CGCTTGCCTTGTCTTCGCATGCATGTGCTCTGTATGTATATAAAAC	fw	8×lex2Op-truncated_pCYC1min
oMM364	GTTTTATATACATACAGAGCACATGCATGCGAAGACAAGGCAAGCG	rev	8×lex2Op-truncated_pCYC1min
oMM408	AAATACACACACTAAATTAATAATGAAAGCGTTAACGGCCAGGC	fw	DEG1t_pCYC1noTATA-LexA
oMM409	GCCTGGCCGTTAACGCTTTCATTATTAATTTAGTGTGTGTATTT	rev	DEG1t_pCYC1noTATA-LexA
oMM540	CTGCGGGCTCTACTTCATCGATCAATAAAGATATCGAGGA	fw	HBD(hER)-B42
oMM541	TCCTCGATATCTTTATTGATCGATGAAGTAGAGCCCGCAG	rev	HBD(hER)-B42
oMM542	GCCTCTTGCTGAGTGGAGATTAACATGTAATTAGTTATGTCACGCT	fw	B42-CYC1t
oMM543	AGCGTGACATAACTAATTACATGTTAATCTCCACTCAGCAAGAGGC	rev	B42-CYC1t
oMM613	TTGACGAGTACGGTGGGTAGCATGTAATTAGTTATGTCAC	fw	VP16-CYC1t
oMM614	GTGACATAACTAATTACATGCTACCCACCGTACTCGTCAA	rev	VP16-CYC1t
oMM655	TGCGGGCTCTACTTCATCGATTCACTTAGACGGCGAGGAC	fw	HBD(hER)-VP16
oMM656	GTCCTCGCCGTCTAAGTGAATCGATGAAGTAGAGCCCGCA	rev	HBD(hER)-VP16
oMM680	TTAACTCGAGATCCCAATGGCGCATGTAATTAGTTATGTCAC		VP64-CYC1t
oMM681	GTGACATAACTAATTACATGCGCCATTGGGATCTCGAGTTAA		VP64-CYC1t
oMM692	CGCGTAATACGACTCACTATAGGGCGAATTGGGTACCAATTGCATGAAGAATCTG	fw	vector- pCMV

oMM800	GAGACCCAAGCTGGCTAGTTATGAAAGCGTTAAC GGCCAG	fw	pCMV-LexA
oMM801	CTGGCCGTTAACGCTTTCATAACTAGCCAGCTTGG GTCTC	rev	pCMV-LexA

Table S5. Primers used in this work for qPCR.

ID	Sequence	Dir.	Description
ot279	CAGGTATTGCCGAAAGAA	fw	Act1
ot280	CCACATTTGTTGGAAGGTA	rev	Act1
ot1062	TGAGGGTGGTGCACAAGATA	fw	Hsp90
ot1063	CCTCTTCAACTGGAGCTTCG	rev	Hsp90
ot1064	CTTGCTGGCAGTGCATAAAA	fw	LexA
ot1065	CTGACGAAGGTCAACGACAA	rev	LexA

DNA Sequences

lex2Op (41 nt)
TGCTGTATATACTCACAGCATAACTGTATATACACCCAGGG

truncated_pCYC1min
GCATGCATGTGCTCTGTATG**TATATAA**AACTCTTGTTTTCTTCTTTTCTCTAAATATTCT
TTCCTTATACATtAGGACCTTTGCAGCATAAATTAC

7×lex2Op(2GT, 37-TATA$_{-52}$)_truncated_pCYC1min (in green: lex2Op sequences)
GAAGACAAGAGCGGAGTTGCTGTATATACTCACAGCATAACTGTATATACACCCAGGG
GTTGCTGTATATACTCACAGCATAACTGTATATACACCCAGGGGTTGCTGTATATACTC
ACAGCATAACTGTATATACACCCAGGGGTTGCTGTATATACTCACAGCATAACTGTATA
TACACCCAGGGGTTGCTGTATATACTCACAGCATAACTGTATATACACCCAGGGGTTG
CTGTATATACTCACAGCATAACTGTATATACACCCAGGGGTTGCTGTATATACTCACAG
CATAACTGTATATACACCCAGGGGCGCTTGCCTTGTCTTCGCATGCATGTcCTCTGTAT
G**TATATAA**AACTCTTGTTTTCTTCTTTTCTCTAAATATTCTTTCCTTATACATtAGGACCT
TTGCAGCATAAATTACa

8×lex2Op(2GT,61-TATA$_{-52}$)_pCYC1min
TTGGAGAGAAGACAAGAGCGGAGTTGCTGTATATACTCACAGCATAACTGTATATACA
CCCAGGGGTTGCTGTATATACTCACAGCATAACTGTATATACACCCAGGGGTTGCTGT
ATATACTCACAGCATAACTGTATATACACCCAGGGGTTGCTGTATATACTCACAGCATA
ACTGTATATACACCCAGGGGTTGCTGTATATACTCACAGCATAACTGTATATACACCCA
GGGGTTGCTGTATATACTCACAGCATAACTGTATATACACCCAGGGGTTGCTGTATATA
CTCACAGCATAACTGTATATACACCCAGGGGTTGCTGTATATACTCACAGCATAACTGT
ATATACACCCAGGGgcGCTTGCCTTGTCTTTCGTGCGACGACACATGATCATATGGCA
TGCATGTGCTCTGTATG**TATATAA**AACTCTTGTTTTCTTCTTTTCTCTAAATATTCTTTC
CTTATACATtAGGACCTTTGCAGCATAAATTACTATACTTCTATAGACACACAAACACAA
ATACACACACTAAATTAATA

8×lex2Op(2GT,37-TATA$_{-52}$)_pCYC1min
GAAGACAAGAGCGGAGTTGCTGTATATACTCACAGCATAACTGTATATACACCCAGGG
GTTGCTGTATATACTCACAGCATAACTGTATATACACCCAGGGGTTGCTGTATATACTC
ACAGCATAACTGTATATACACCCAGGGGTTGCTGTATATACTCACAGCATAACTGTATA
TACACCCAGGGGTTGCTGTATATACTCACAGCATAACTGTATATACACCCAGGGGTTG
CTGTATATACTCACAGCATAACTGTATATACACCCAGGGGTTGCTGTATATACTCACAG
CATAACTGTATATACACCCAGGGGTTGCTGTATATACTCACAGCATAACTGTATATACAC
CCAGGGGCGCTTGCCTTGTCTTCGCATGCATGTGCTCTGTATG**TATATAA**AACTCTTG
TTTTCTTCTTTTCTCTAAATATTCTTTCCTTATACATtAGGACCTTTGCAGCATAAATTAC
TATACTTCTATAGACACACAAACACAAATACACACACTAAATTAATAATG

8×lex2Op(2GT,84-TATA$_{-106}$,138$_{-}$-TATA$_{-52}$)_pCYC1core

GAAGACCAAGAGCGGAGTTGCTGTATATACTCACAGCATAACTGTATATACACCCAGG
GGTTGCTGTATATACTCACAGCATAACTGTATATACACCCAGGGGTTGCTGTATATACT
CACAGCATAACTGTATATACACCCAGGGGTTGCTGTATATACTCACAGCATAACTGTAT
ATACACCCAGGGGTTGCTGTATATACTCACAGCATAACTGTATATACACCCAGGGGTT
GCTGTATATACTCACAGCATAACTGTATATACACCCAGGGGTTGCTGTATATACTCACA
GCATAACTGTATATACACCCAGGGGTTGCTGTATATACTCACAGCATAACTGTATATACA
CCCAGGGGCGCTTGCCTTGTCTTCCAGATCCGCCAGGCGTGTATATATAGCGTGGAT
GGCCAGGCAACTTTAGTGCTGACACATACAGGCATATATATATGTGTGCGACGACACA
TGATCATATGGCATGCATGTGCTCTGTATG**TATATAA**AACTCTTGTTTTCTTCTTTTCTC
TAAATATTCTTTCCTTATACATtAGGACCTTTGCAGCATAAATTACTATACTTCTATAGAC
ACACAAACACAAATACACACACTAAATTAATA

lex2Op(6-TATA$_{-106}$,60-TATA$_{-52}$)_pCYC1core
CAGATCCGCCAGGCGTGTATATATAGCGTGGATGGCCAGGCAACTTTAGTGCTGACA
CATATGCTGTATATACTCACAGCATAACTGTATATACACCCAGGGCAGGCATATATATAT
GTGTGCGACGACACATGATCATATGGCATGCATGTGCTCTGTATG**TATATAA**AACTCTT
GTTTTCTTCTTTTCTCTAAATATTCTTTCCTTATACATtAGGACCTTTGCAGCATAAATTA
CTATACTTCTATAGACACACAAACACAAATACACACACTAAATTAATA

lexOp2(37-TATA$_{-52}$)_pCYC1core
CAGATCCGCCAGGCGTGTATATATAGCGTGGATGGCCAGGCAACTTTAGTGCTGACA
CATACAGGCATATATATATGTGTGCGATGCTGTATATACTCACAGCATAACTGTATATAC
ACCCAGGGCGACACATGATCATATGGCATGCATGTGCTCTGTATG**TATATAA**AACTCT
TGTTTTCTTCTTTTCTCTAAATATTCTTTCCTTATACATtAGGACCTTTGCAGCATAAATT
ACTATACTTCTATAGACACACAAACACAAATACACACACTAAATTAATA

lexOp2(10-TATA$_{-52}$)_pCYC1core
CAGATCCGCCAGGCGTGTATATATAGCGTGGATGGCCAGGCAACTTTAGTGCTGACA
CATACAGGCATATATATATGTGTGCGACGACACATGATCATATGGCATGCATGTGCTCT
GCTGTATATACTCACAGCATAACTGTATATACACCCAGGGGCTCTGTATG**TATATAA**AA
CTCTTGTTTTCTTCTTTTCTCTAAATATTCTTTCCTTATACATtAGGACCTTTGCAGCATA
AATTACTATACTTCTATAGACACACAAACACAAATACACACACTAAATTAATA

lexOp2(6-TATA$_{-52}$)_pCYC1core
CAGATACGCCAGGCGTGTATATATAGCGTGGATGGCCAGGCAACTTTAGTGCTGACA
CATACAGGCATATATATATGTGTGCGACGACACATGATCATATGGCATGCATGTGCTCT
GCTGTATATACTCACAGCATAACTGTATATACACCCAGGGTGTATG**TATATAA**AACTCTT
GTTTTCTTCTTTTCTCTAAATATTCTTTCCTTATACATtAGGACCTTTGCAGCATAAATTA
CTATACTTCTATAGACACACAAACACAAATACACACACTAAATTAATA

2×lex2Op(2-GT,138-TATA$_{-52}$)_pCYC1core
GAGAAGACAAGAGCGGAGTTGCTGTATATACTCACAGCATAACTGTATATACACCCAG
GGGTTGCTGTATATACTCACAGCATAACTGTATATACACCCAGGGGCGCTTGCCTTGT

CTTCCAGATCCGCCAGGCGTGTATATATAGCGTGGATGGCCAGGCAACTTTAGTGCT
GACACATACAGGCATATATATATGTGTGCGACGACACATGATCATATGGCATGCATGTG
CTCTGTATG**TATATAA**AACTCTTGTTTTCTTCTTTTCTCTAAATATTCTTTCCTTATACATt
AGGACCTTTGCAGCATAAATTACTATACTTCTATAGACACACAAACACAAATACACACA
CTAAATTAATA

2×lex2Op(15,60-TATA$_{-52}$)_pCYC1core
CAGATCCGCCAGGCGTGTATATATAGCGTGGATGGCCAGGCAACTTTGCTGTATATAC
TCACAGCATAACTGTATATACACCCAGGGTAGTGCTGACACATATGCTGTATATACTCA
CAGCATAACTGTATATACACCCAGGGCAGGCATATATATATGTGTGCGACGACACATGA
TCATATGGCATGCATGTGCTCTGTATG**TATATAA**AACTCTTGTTTTCTTCTTTTCTCTAA
ATATTCTTTCCTTATACATtAGGACCTTTGCAGCATAAATTACTATACTTCTATAGACACA
CAAACACAAATACACACACTAAATTAATA

2×lex2Op(42,60-TATA$_{-52}$)_pCYC1core
CAGATCCGCCAGGCGTGTATGCTGTATATACTCACAGCATAACTGTATATACACCCAG
GGTATATAGCGTGGATGGCCAGGCAACTTTAGTGCTGACACATATGCTGTATATACTC
ACAGCATAACTGTATATACACCCAGGGCAGGCATATATATATGTGTGCGACGACACATG
ATCATATGGCATGCATGTGCTCTGTATG**TATATAA**AACTCTTGTTTTCTTCTTTTCTCTA
AATATTCTTTCCTTATACATtAGGACCTTTGCAGCATAAATTACTATACTTCTATAGACAC
ACAAACACAAATACACACACTAAATTAATA

3×lex2Op(15,60-TATA$_{-52}$)_pCYC1core
CAGATCCGCCAGGCGTGTATATATAGCGTGGTGCTGTATATACTCACAGCATAACTGTA
TATACACCCAGGGATGGCCAGGCAACTTTGCTGTATATACTCACAGCATAACTGTATAT
ACACCCAGGGTAGTGCTGACACATATGCTGTATATACTCACAGCATAACTGTATATACA
CCCAGGGCAGGCATATATATATGTGTGCGACGACACATGATCATATGGCATGCATGTG
CTCTGTATG**TATATAA**AACTCTTGTTTTCTTCTTTTCTCTAAATATTCTTTCCTTATACATt
AGGACCTTTGCAGCATAAATTACTATACTTCTATAGACACACAAACACAAATACACACA
CTAAATTAATA

3×lex2Op(2-GT,138-TATA$_{-52}$)_pCYC1core
GAGAAGACAAGAGCGGAGTTGCTGTATATACTCACAGCATAACTGTATATACACCCAG
GGGTTGCTGTATATACTCACAGCATAACTGTATATACACCCAGGGGTTGCTGTATATAC
TCACAGCATAACTGTATATACACCCAGGGGCGCTTGCCTTGTCTTCCAGATCCGCCA
GGCGTGTATATATAGCGTGGATGGCCAGGCAACTTTAGTGCTGACACATACAGGCATA
TATATATGTGTGCGACGACACATGATCATATGGCATGCATGTGCTCTGTATG**TATATAA**
AACTCTTGTTTTCTTCTTTTCTCTAAATATTCTTTCCTTATACATtAGGACCTTTGCAGCA
TAAATTACTATACTTCTATAGACACACAAACACAAATACACACACTAAATTAATA

3×lex2Op(6,60-TATA$_{-52}$)_pCYC1core
CAGATCCGCCAGGCGTGTATATATAGCGTGGATGGCCAGGCAACTTTAGTGCTGTATA
TACTCACAGCATAACTGTATATACACCCAGGGTGCTGATGCTGTATATACTCACAGCAT
AACTGTATATACACCCAGGGCACATATGCTGTATATACTCACAGCATAACTGTATATACA

CCCAGGGCAGGCATATATATATGTGTGCGACGACACATGATCATATGGCATGCATGTG
CTCTGTATG**TATATAA**AACTCTTGTTTTCTTCTTTTCTCTAAATATTCTTTCCTTATACATt
AGGACCTTTGCAGCATAAATTACTATACTTCTATAGACACACAAACACAAATACACACA
CTAAATTAATA

3×lex2Op(21,60-TATA$_{-52}$)_pCYC1core
CAGATCCGCCAGGCGTGTATGCTGTATATACTCACAGCATAACTGTATATACACCCAG
GGTATATAGCGTGGATGGCCAGGTGCTGTATATACTCACAGCATAACTGTATATACACC
CAGGGCAACTTTAGTGCTGACACATATGCTGTATATACTCACAGCATAACTGTATATAC
ACCCAGGGCAGGCATATATATATGTGTGCGACGACACATGATCATATGGCATGCATGT
GCTCTGTATG**TATATAA**AACTCTTGTTTTCTTCTTTTCTCTAAATATTCTTTCCTTATACA
TtAGGACCTTTGCAGCATAAATTACTATACTTCTATAGACACACAAACACAAATACACAC
ACTAAATTAATA

3×lex2Op(42,60-TATA$_{-52}$)_pCYC1core
GCGCCAGTTCATTTGGCGAGCGTTGGTTGGTGGATCAAGTGCTGTATATACTCACAG
CATAACTGTATATACACCCAGGGCCCACGCGTAGGCAATCCTCGAGCAGATCCGCCA
GGCGTGTATGCTGTATATACTCACAGCATAACTGTATATACACCCAGGGTATATAGCGT
GGATGGCCAGGCAACTTTAGTGCTGACACATATGCTGTATATACTCACAGCATAACTG
TATATACACCCAGGGCAGGCATATATATATGTGTGCGACGACACATGATCATATGGCAT
GCATGTGCTCTGTATG**TATATAA**AACTCTTGTTTTCTTCTTTTCTCTAAATATTCTTTCC
TTATACATtAGGACCTTTGCAGCATAAATTACTATACTTCTATAGACACACAAACACAAAT
ACACACACTAAATTAATA

3×lex2Op(2,60-TATA$_{-52}$)_pCYC1core
CAGATCCGCCAGGCGTGTATATATAGCGTGGATGGCCAGGCAACTTTAGTGCTGACA
TGCTGTATATACTCACAGCATAACTGTATATACACCCAGGGCATGCTGTATATACTCAC
AGCATAACTGTATATACACCCAGGGTATGCTGTATATACTCACAGCATAACTGTATATAC
ACCCAGGGCAGGCATATATATATGTGTGCGACGACACATGATCATATGGCATGCATGT
GCTCTGTATG**TATATAA**AACTCTTGTTTTCTTCTTTTCTCTAAATATTCTTTCCTTATACA
TtAGGACCTTTGCAGCATAAATTACTATACTTCTATAGACACACAAACACAAATACACAC
ACTAAATTAATA

lex2Op(60-TATA$_{-52}$)_truncated_pCYC1core
CAGATCCGCCAGGCGTGTATATATAGCGTGGATGGCCAGGCAACTTTAGTGCTGACA
CATATGCTGTATATACTCACAGCATAACTGTATATACACCCAGGGCAGGCATATATATAT
GTGTGCGACGACACATGATCATATGGCATGCATGTGCTCTGTATG**TATATAA**AACTCTT
GTTTTCTTCTTTTCTCTAAATATTCTTTCCTTATACATtAGGACCTTTGCAGCATAAATTA
C

3×lex2Op(15,60-TATA$_{-52}$)_truncated_pCYC1core
CAGATCCGCCAGGCGTGTATATATAGCGTGGTGCTGTATATACTCACAGCATAACTGTA
TATACACCCAGGGATGGCCAGGCAACTTTGCTGTATATACTCACAGCATAACTGTATAT

ACACCCAGGGTAGTGCTGACACATATGCTGTATATACTCACAGCATAACTGTATATACA
CCCAGGGCAGGCATATATATATGTGTGCGACGACACATGATCATATGGCATGCATGTG
CTCTGTATG**TATATAA**AACTCTTGTTTTCTTCTTTTCTCTAAATATTCTTTCCTTATACATt
AGGACCTTTGCAGCATAAATTAC

pGPD
cagttcgagtttatcattatcaatactgccatttcaaagaatacgtaaataattaatagtagtgattttcctaactttatttagtcaaa
aaattagccttttaattctgctgtaacccgtacatgcccaaaatagggggcgggttacacagaatatataacatcgtaggtgtc
tgggtgaacagtttattcctggcatccactaaatataatggagcccgctttttaagctggcatccagaaaaaaaaagaatccc
agcaccaaaatattgttttcttcaccaaccatcagttcataggtccattctcttagcgcaactacagagaacaggggcacaaa
caggcaaaaaacgggcacaacctcaatggagtgatgcaacctgcctggagtaaatgatgacacaaggcaattgaccca
cgcatgtatctatctcattttcttacaccttctattaccttctgctctctctgatttggaaaaagctgaaaaaaaaggttgaaaccag
ttccctgaaattattcccctacttgactaataagtatataaagacggtaggtattgattgtaattctgtaaatctatttcttaaacttctt
aaattctacttttatagttagtcttttttttagttttaaaacaccaagaacttagtttcgaataaacacacataaacaaacaaaA

DEG1t_pCYC1noTATA
AATAATATATAAACCTGTATAATATAACCTTGAAGACTATATTTCTTTTCttcTTTCCTTATAC
ATtAGGACCTTTGCAGCATAAATTACTATACTTCTATAGACACACAAACACAAATACACA
CACTAAATTAATA

pCMV
AATTGCATGAAGAATCTGCTTAGGGTTAGGCGTTTTGCGCTGCTTCGCGATGTACGG
GCCAGATATACGCGTTGACATTGATTATTGACTAGTTATTAATAGTAATCAATTACGGGG
TCATTAGTTCATAGCCCATATATGGAGTTCCGCGTTACATAACTTACGGTAAATGGCCC
GCCTGGCTGACCGCCCAACGACCCCCGCCCATTGACGTCAATAATGACGTATGTTCC
CATAGTAACGCCAATAGGGACTTTCCATTGACGTCAATGGGTGGACTATTTACGGTAA
ACTGCCCACTTGGCAGTACATCAAGTGTATCATATGCCAAGTACGCCCCCTATTGACG
TCAATGACGGTAAATGGCCCGCCTGGCATTATGCCCAGTACATGACCTTATGGGACTT
TCCTACTTGGCAGTACATCTACGTATTAGTCATCGCTATTACCATGGTGATGCGGTTTT
GGCAGTACATCAATGGGCGTGGATAGCGGTTTGACTCACGGGGATTTCCAAGTCTCC
ACCCCATTGACGTCAATGGGAGTTTGTTTTGGCACCAAAATCAACGGGACTTTCCAA
AATGTCGTAACAACTCCGCCCCATTGACGCAAATGGGCGGTAGGCGTGTACGGTGG
GAGGTCTATATAAGCAGAGCTCTCTGGCTAACTAGAGAACCCACTGCTTACTGGCTTA
TCGAAATTAATACGACTCACTATAGGGAGACCCAAGCTGGCTAGTT

yEGFP
ATGTCTAAAGGTGAAGAATTATTCACTGGTGTTGTCCCAATTTTGGTTGAATTAGATGG
TGATGTTAATGGTCACAAATTTTCTGTCTCCGGTGAAGGTGAAGGTGATGCTACTTAC
GGTAAATTGACCTTAAAATTTATTTGTACTACTGGTAAATTGCCAGTTCCATGGCCAAC
CTTAGTCACTACTTTCGGTTATGGTGTTCAATGTTTTGCGAGATACCCAGATCATATGA
AACAACATGACTTTTTCAAGTCTGCCATGCCAGAAGGTTATGTTCAAGAAAGAACTAT
TTTTTTCAAAGATGACGGTAACTACAAGACCAGAGCTGAAGTCAAGTTTGAAGGTGAT
ACCTTAGTTAATAGAATCGAATTAAAAGGTATTGATTTTAAAGAAGATGGTAACATTTTA
GGTCACAAATTGGAATACAACTATAACTCTCACAATGTTTACATCATGGCTGACAAACA

AAAGAATGGTATCAAAGTTAACTTCAAAATTAGACACAACATTGAAGATGGTTCTGTTC
AATTAGCTGACCATTATCAACAAAATACTCCAATTGGTGATGGTCCAGTCTTGTTACCA
GACAACCATTACTTATCCACTCAATCTGCCTTATCCAAAGATCCAAACGAAAAGAGAG
ACCACATGGTCTTGTTAGAATTTGTTACTGCTGCTGGTATTACCCATGGTATGGATGAA
TTGTACAAATAA

CYC1t
CATGTAATTAGTTATGTCACGCTTACATTCACGCCCTCCCCCCACATCCGCTCTAACC
GAAAAGGAAGGAGTTAGACAACCTGAAGTCTAGGTCCCTATTTATTTTTTTATAGTTAT
GTTAGTATTAAGAACGTTATTTATATTTCAAATTTTTCTTTTTTTTCTGTACAGACGCGTG
TACGCATGTAACATTATACTGAAAACCTTGCTTGAGAAGGTTTTGGGACGCTCGAAGG
CTTTAATTTGCAAGCTatc

Biosensor schemes

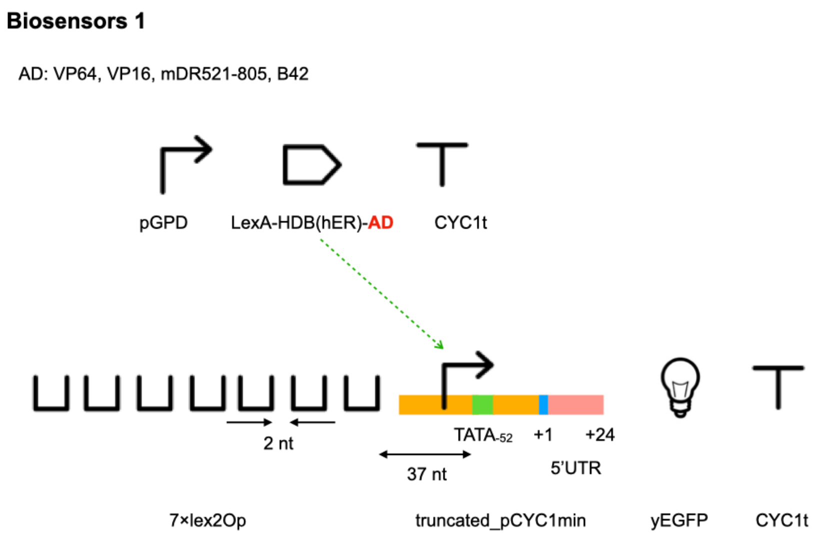

Figure S6. General scheme of biosensors 1.

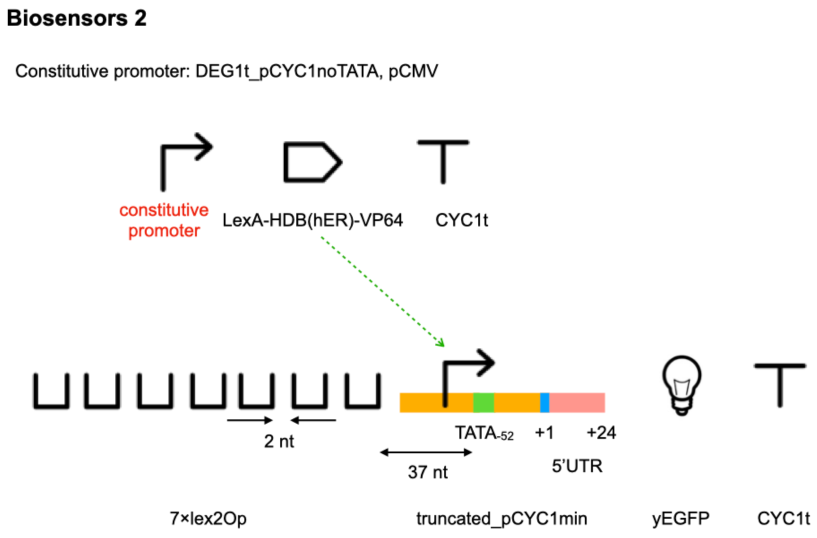

Figure S7. General scheme of biosensors 2.

Biosensor 3 (byMM1376)

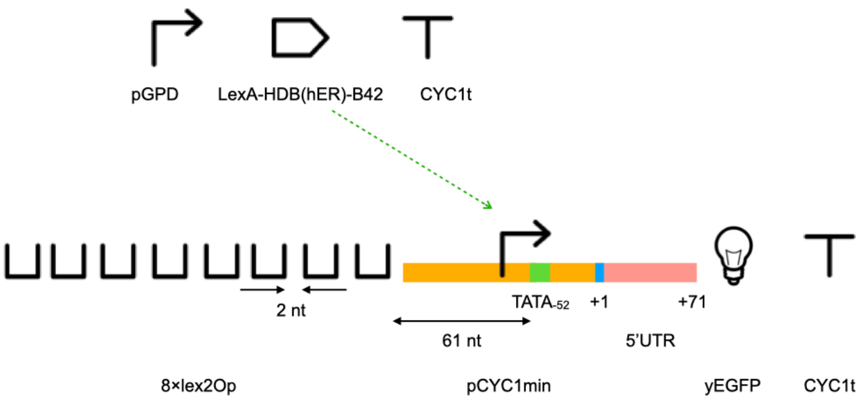

Figure S8. Biosensor 3 as engineered in the strain byMM1376.

Biosensors 4 (byMM382)

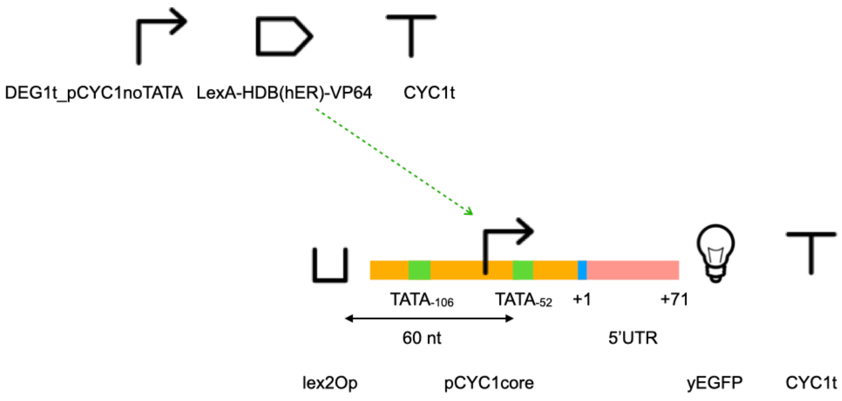

Figure S9. Biosensor 4 as engineered in the strain byMM382.

Biosensors 5

Constitutive promoter and AD pairs: pGPD-B42, pCMV-VP64

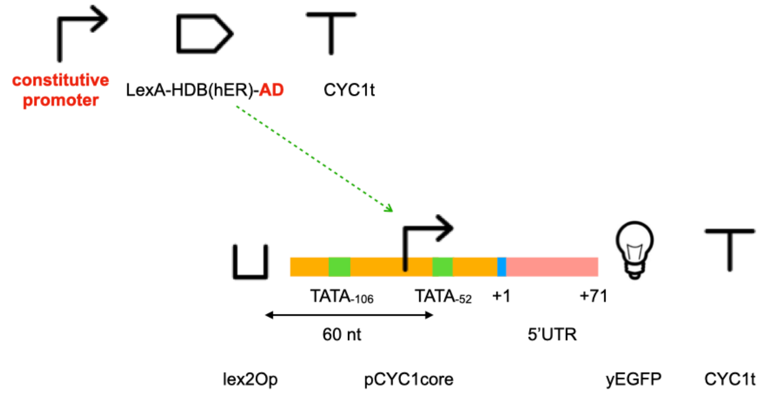

Figure S10. General scheme of biosensors 5.

Biosensors 6 (byMM482)

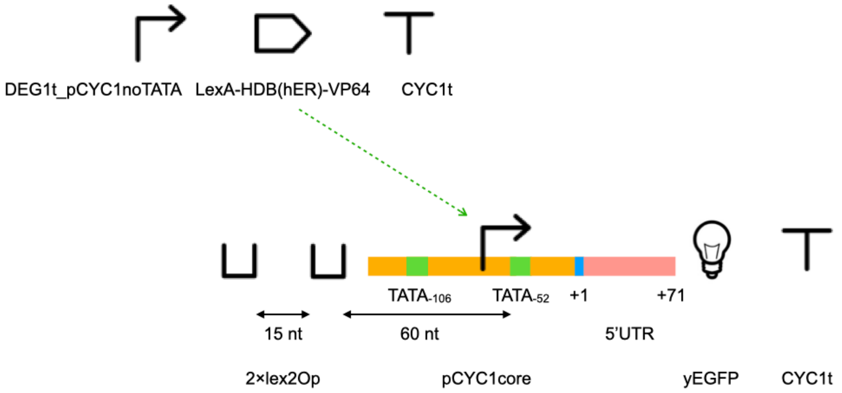

Figure S11. Biosensor 6 as engineered in the strain byMM482.

Biosensors 7 (byMM381)

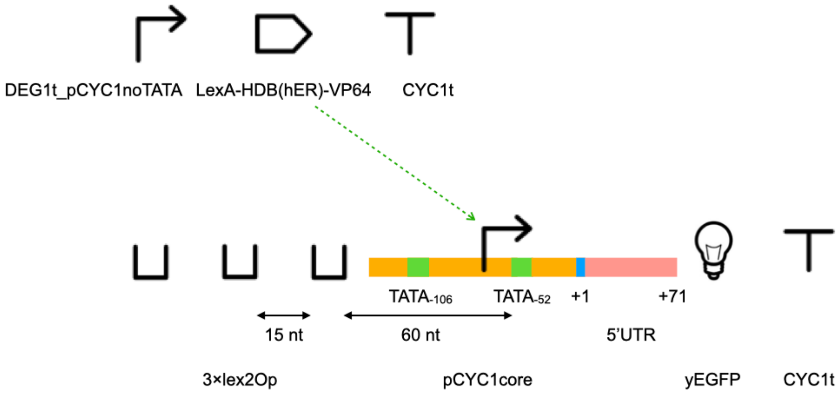

Figure S12. Biosensor 7 as engineered in the strain byMM381.

Biosensors 8 (byMM496)

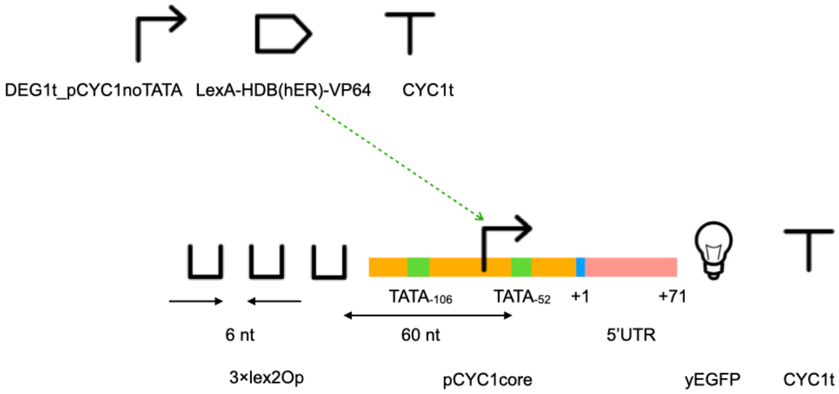

Figure S13. Biosensor 8 as engineered in the strain byMM496.

Biosensors 9 (byMM367)

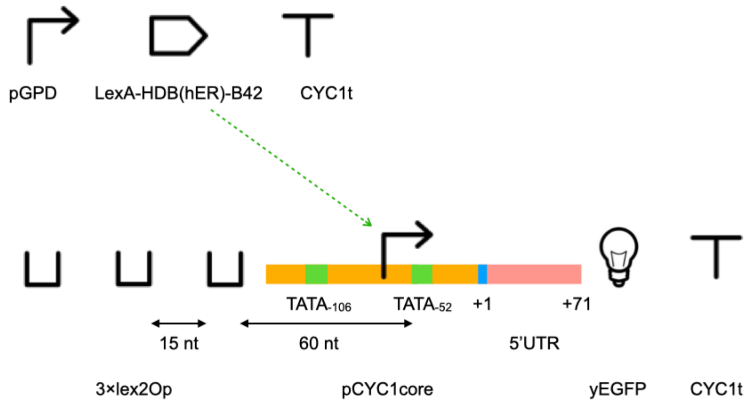

Figure S14. Biosensor 9 as engineered in the strain byMM367.

Biosensors 10 (byMM1448)

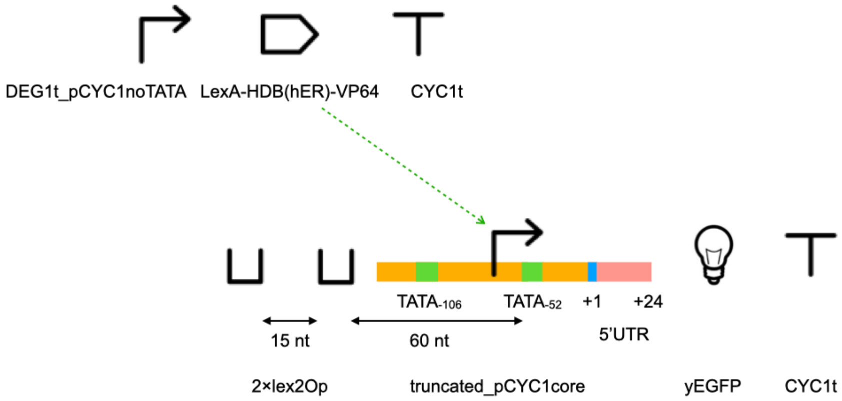

Figure S15. Biosensor 10 as engineered in the strain byMM1448.
